# Self-recoverable mechanoluminescence in simple oxides: Al_2_O_3_:Cr

**DOI:** 10.1038/s41377-026-02274-w

**Published:** 2026-04-15

**Authors:** Ziyi Fang, Xiaofeng Pan, Qi’an Zhang, Mingzhi Wu, Yang Liu, Qidong Ma, Biyun Ren, Yanze Wang, Shengqiang Liu, Maryam Zulfiqar, Ming-Gang Ju, Jiulin Gan, Leipeng Li, Feng Wang, Dengfeng Peng

**Affiliations:** 1 College of Physics and Optoelectronic Engineering, Shenzhen University, Shenzhen, 518060 China; 2 Key Laboratory of Quantum Materials and Devices of Ministry of Education, School of Physics, Southeast University, Nanjing, 21189 China; 3 State Key Laboratory of Luminescent Material and Devices, and Guangdong Provincial Key Laboratory of Fibre Laser Materials and Applied Techniques, Guangdong Engineering Technology Research and Development Center of Special Optical Fiber Materials and Devices, South China University of Technology, Guangzhou, 510641 China; 4 College of Physics Science and Technology, Hebei University, Baoding, China; 5 Department of Materials Science and Engineering, City University of Hong Kong, Hong Kong SAR, China; 6 Hong Kong Institute for Clean Energy, City University of Hong Kong, Hong Kong SAR, China; 7 Key Laboratory of Optoelectronic Devices and Systems of Ministry of Education and Guangdong Province,Shenzhen University, Shenzhen, Guangdong, 518060 China; 8 China State Key Laboratory of Radio Frequency Heterogeneous Integration, Shenzhen University, Shenzhen, Guangdong, 518060 China

**Keywords:** Optical materials and structures, Optical physics

## Abstract

Materials exhibiting mechanoluminescence (ML) that directly convert mechanical stimuli into light hold significant potential for real-time stress sensing and intelligent photonic systems. However, most high-performance ML systems rely on complex multicomponent compounds that often suffer from limited intensity, stability, and scalability, largely due to poorly understood mechanisms. Herein, we report a simple Al_2_O_3_:Cr^3+^ oxide that exhibits unprecedented ML intensity, enabled by a well-defined mechanical-to-optical energy conversion process. The self-recoverable ML arises from stress-induced ionization of electrons from luminescence centers, followed by their recapture upon stress release. By precisely tuning the doping levels, annealing conditions, and heterojunction interfaces, Al_2_O_3_:Cr^3+^ phosphors achieved intense, reproducible, and thermally stable near-infrared emission. Notably, high-temperature annealing dramatically enhanced the ML intensity, with thermodynamic and kinetic analyses revealing increases in the carrier and defect concentrations by several orders of magnitude, accounting for the exceptional brightness. By leveraging the chemical robustness, abundance, and low cost of alumina, we demonstrated the flexible ML paper for stress visualization and multi-level anti-counterfeiting, as well as in-situ grown Al_2_O_3_:Cr^3+^ luminescent layers on Cr–Al alloys for passive, real-time stress monitoring. This study establishes Al_2_O_3_ as a durable and scalable oxide platform for next-generation self-recoverable ML materials, bridging fundamental research and practical sensing technologies.

## Abstract (web summary)

A low-cost Al_2_O_3_:Cr^3+^ platform exhibits self-recoverable mechanoluminescence (ML) via reversible Cr charge transitions. Defect engineering and Al_2_O_3_/Ga_2_O_3_ heterojunctions produce strong ML for real-time stress visualization.

## Graphical abstract

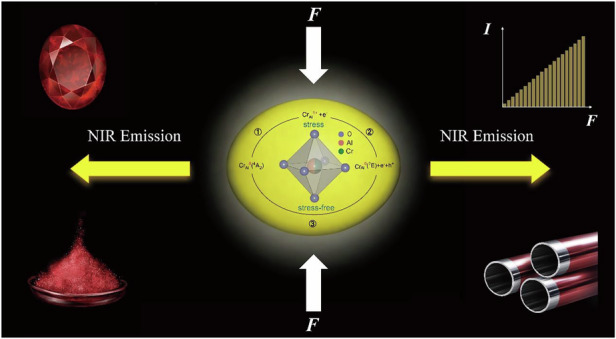

## Introduction

Mechanoluminescence (ML) refers to the phenomenon in which a material emits light in response to mechanical stimuli such as fracture^1^, friction^2^, compression^3^, tension^4^, or ultrasound^5,6^. Unlike conventional electroluminescence, photoluminescence (PL)^7^, and chemiluminescence, ML enables the direct conversion of mechanical energy into light without requiring any external energy input source^8,9^. Owing to their real-time optical response, ML materials hold significant promise for applications in stress sensing and visualization^10–12^, multi-level anti-counterfeiting^13,14^, and biomedical technologies^15–17^. Current research on ML has been primarily focused on materials that emit in the ultraviolet (UV) to visible spectral range, such as SrAl_2_O_4_:Eu^18^, ZnS:Mn/Cu^19–23^, CaZnOS:Mn^24^, SrZnOS:Mn^1^, BaSi_2_O_2_N_2_:Eu^25^, Sr_2_P_2_O_7_:Pr^26^, MgF_2_:Mn^27^, CaF_2_:Tb^4^, SrF_2_:Pr^3^, and AlN^28^. However, visible light is susceptible to environmental scattering and background interference, which limits its effectiveness in complex environments. By contrast, near-infrared (NIR) ML materials offer greater penetration depth and reduced visible-light interference, making them well suited for specialized applications such as long-range detection, internal stress monitoring of structures, and tissue bioimaging. In parallel with these application demands, increasing attention has been paid to self-recoverable ML systems, in which luminescence can be repeatedly triggered under cyclic mechanical stimulation without external recharging. Several representative systems exhibiting cyclic ML responses have been reported in recent years, such as Sr_3_(BO_3_)_2_:Pr^8^ and SrZnOSe:Nd^15^. Nevertheless, NIR ML systems remain limited in number and are frequently non–self-recovering, relying on UV pre-excitation to generate trap-controlled emission (e.g., LiNbO_3_:Nd^29^) or on irreversible fractoluminescence^30^. These mechanisms suffer from delayed response and lack of self-recovery, significantly restricting their utility in dynamic or real-time monitoring applications. Therefore, the development of novel NIR ML materials featuring low cost, self-recoverability, and high emission efficiency is essential for advancing their practical applications.

Despite the growing interest in ML, its underlying mechanisms remain incompletely understood. Identifying specific defect states and probing ultrafast carrier dynamics under mechanical stress pose significant experimental challenges, causing most ML advancements to rely on empirical trial-and-error approaches. Consequently, the diversity of ML systems remains limited, and their development trails behind that of more established luminescent technologies. Current theoretical efforts primarily emphasize the calculation of defect formation energies and post-doping electronic structures; however, the pivotal question of how external stress modulates charge-carrier behavior and emission processes has been inadequately explored. Owing to its unique [Ar]3d^3^ electronic configuration, Cr^3+^ exhibits exceptional luminescent properties in the NIR region. Its emission spectrum includes sharp R-line emission (^2^E → ^4^A_2_, ~700 nm) and broad-band emission (^4^T_2_ → ^4^A_2_, ~750–1200 nm), with the emission wavelength tunable via the modulation of the local crystal field^31^. This electronic configuration renders Cr^3+^ particularly effective for NIR emission because its luminescence primarily arises from d–d transitions, which exhibit relatively high transition probabilities in the NIR range, thereby enabling efficient light emission. Compared with trivalent rare-earth ions, Cr^3+^ ions possess the advantages of strong light absorption, broad absorption range, and continuously tunable emission peaks^32^. In recent years, Cr^3+^ has garnered significant attention in the design of ML materials (e.g., LiGa_5_O_8_^33^, ZnGa_2_O_4_^34^, Ga_2_O_3_^13,35^, MgGa_2_O_4_^36^, SrGa_12_O_19_^34^, Gd_3_Ga_5_O_12_^37^, MgO^38,39^, and LaAlO_3_^40^). Nonetheless, despite their promising performance, these materials are constrained by high production costs and complex crystal structures, which substantially limit their large-scale application and further commercialization. By contrast, Al_2_O_3_ offers a structurally simple, earth-abundant, and industrially mature oxide host, yet its ML has remained largely unexplored compared with complex gallate- or garnet-based systems.

In this study, we present Al_2_O_3_:Cr^3+^ as a simple and durable oxide platform that delivers unprecedented ML intensity and practical usability. Its self-recoverable emission mechanism was clarified via theoretical calculations and experiments; ML self-recovery relies on reversible Cr_Al_^0^/Cr_Al_^1+^ charge transitions, whereas NIR emission originates from radiative transitions between the ^2^E to ^4^A_2_ states of Cr^3+^. We synthesized Al_2_O_3_:Cr^3+^ phosphors using low-cost, high-temperature solid-state methods, optimizing the Cr^3+^ concentration and annealing conditions. High-temperature annealing significantly enhances carrier and defect concentrations by orders of magnitude, yielding intense, stable, and thermally robust ML. Further optimization was performed using Al_2_O_3_/Ga_2_O_3_:Cr^3+^ heterojunctions to enable synergistic defect and ML modulation. Leveraging the robustness, abundance, and scalability of alumina, we extended the material to two applications: (1) embedding Al_2_O_3_/Ga_2_O_3_:Cr^3+^ powders into cellulose pulp for flexible NIR ML paper for invisible handwriting, stress mapping, anti-counterfeiting; and (2) performing in situ thermal oxidation of Cr–Al alloys to generate rigid Al_2_O_3_:Cr^3+^ layers for real-time, excitation-free engineering stress visualization.

## Results

**Fig. 1:**
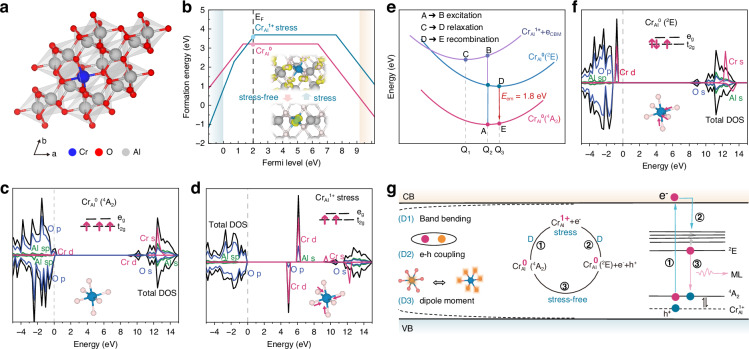
Theoretical investigation of self-recoverable ML emission in Al_2_O_3_:Cr^3+^. **a** Crystal structure of Al_2_O_3_:Cr^3+^. **b** Calculated formation energies of Cr_Al_ defects under stress-free and stressed conditions. Self-consistent Fermi levels are indicated by vertical dashed lines. Local atomic structure: Cr atoms (blue spheres) and charge density distribution (yellow isosurfaces). **c** Electronic densities of states (DOS) for the ground-state Cr_Al_^0^(^4^A_2_) defect under stress-free conditions, and the corresponding CrO_6_ octahedral structure (blue sphere: Cr; red sphere: O). Arrow diagram showing three electrons of Cr occupying the t_2g_ energy level. **d** Electronic DOS for the ionized-state Cr_Al_^1+^ defect under stressed conditions, and the corresponding CrO_6_ octahedral structure (blue sphere: Cr; red sphere: O). Arrow diagram showing two electrons of Cr occupying the t_2g_ energy level. **e** Schematic diagram of the transitions between different states during electron excitation, relaxation, and recombination in Al_2_O_3_:Cr^3+^ in the ligand field. **f** Electronic DOS for the excited-state Cr_Al_^0^(^2^E) defect under stress-free conditions, and the corresponding CrO_6_ octahedral structure (blue sphere: Cr; red sphere: O). Arrow diagram showing three electrons of Cr occupying the t_2g_ energy level, one with opposite spin. **g** Upon strain excitation, bound excitons stored in activators are ionized and excited to CBM (process 1), followed by relaxation (process 2) and radiative recombination between electrons in the excited states and holes in the ground state (process 3). The driving forces (denoted as D) in this process include band bending, enhanced dipole moment, and enhanced electron–phonon coupling

Al_2_O_3_ exhibited a direct bandgap of 8.61 eV, consistent with the experimentally measured value of 8.70 eV and demonstrated its intrinsic insulating characteristics (Fig. S1)^41^. With Cr doping, the atoms tended to substitute Al, forming Cr_Al_ defects with CrO_6_ octahedra (Fig. S2a), as illustrated in Fig. 1a. To investigate the stress response mechanism underlying the ML process, we systematically calculated the formation energies of all intrinsic and extrinsic point defects in Al_2_O_3_:Cr^3+^ under both stressed and stress-free conditions (Figs. S2, S3). The phase stability of Al_2_O_3_:Cr^3+^ was determined by calculating the total energies of competing phases within the Al–O–Cr chemical space (see Supplementary Information). Considering the experimental synthesis conditions, the formation energies of all defects were calculated under O-rich equilibrium growth conditions. Notably, only minimal changes were observed in the formation energies (Fig. S2b, c) and transition levels (Fig. S2d, e) of most defects under stress. Interestingly, the Cr_Al_ defect—which serves as the dominant active center in the Al_2_O_3_:Cr^3+^ system—exhibited subtle changes under stress. To determine the stable form of defects under specific synthesis conditions, the evolution of the self-consistent Fermi level (E_F_) with growth conditions is shown in Fig. S2f. Remarkably, the Cr_Al_ defect was preferentially stabilized in a neutral state (Cr_Al_^0^) under stress-free conditions, whereas applied stress induced a valence transition to the +1 charged state (Cr_Al_^1+^), demonstrating a stress-driven ionization mechanism (Fig. 1b). Compared with pristine Al_2_O_3_, the neutral state of Cr_Al_^0^ created relatively shallow spin-up and spin-down defect states adjacent to the valence band maximum (VBM) and conduction band minimum (CBM), respectively (Fig. 1c). Under applied strain, Cr_Al_^1+^ produced distinct spin-polarized impurity states within the bandgap (Fig. 1d). In addition, Cr_Al_^1+^ exhibited a Jahn–Teller distortion, in which the bond length is typically contracted compared with the ground-state Cr_Al_^0^ configuration (insets in Fig. 1c, d). Upon stress release, the ionized electrons undergo detrapping and recombination, thereby repopulating the active centers and restoring the system to Cr_Al_^0^, which accounts for the observed self-recoverable ML behavior.

To illustrate the luminescence process involving different electronic states, Fig. 1e depicts a schematic of the transitions between different states during electron excitation, relaxation, and recombination in Al_2_O_3_:Cr^3+^. In particular, the 3d^3^ configuration of Cr^3+^ induced by the ligand field was split into e_g_ and t_2g_ energy levels^42,43^, corresponding to the formation of ground-state Cr_Al_^0^(^4^A_2_) (inset in Fig. 1c) and low-energy excited-state Cr_Al_^0^(^2^E) (inset in Fig. 1f). Structural analysis showed that, compared with the ground state, the ^2^E excited state exhibited obvious Jahn–Teller distortion, characterized by compressed equatorial bond lengths. The density of states (DOS) further indicated that the ^2^E excited state exhibited stronger occupied states near the Fermi level, reflecting stronger interactions within the CrO_6_ unit in the excited state and resulting in an overall higher energy (Fig. 1f). Under applied stress, the ionized-state Cr_Al_^1+^ remained stable. However, upon stress removal, Cr_Al_^1+^ readily recaptured an electron, converting it into the neutral- and excited-state Cr_Al_^0^(^2^E), which subsequently underwent radiative recombination to return to the ground state (Fig. 1e). Therefore, the observed luminescence stemmed from electron transition between the excited and ground states (^2^E → ^4^A_2_)^44^. The theoretical transition energies calculated using constrained density functional theory (cDFT) and the ΔSCF method were determined to be 1.8 eV after functional correction, showing an excellent agreement with the experimental result of 696 nm (1.79 eV)^44^.

This work further systematically investigated the charge redistribution and band-structure modulation in Al_2_O_3_:Cr^3+^ systems under external stimuli. Analysis of the average charge density difference (Δρ) and electrostatic potential profiles between the ground and excited states (Fig. S4) demonstrated stress-induced charge depletion (~ 0.2 e/Å) and potential change (~ 0.15 eV) around Cr^3+^ sites, accompanied by enhanced dipole moments. This pronounced charge separation, comparable to that observed in transition-metal dichalcogenides^45^, establishes the fundamental driving force for electron migration. Combined non-adiabatic molecular dynamics (NAMD) and DFT calculations revealed strong electron–phonon coupling during ionization. Fourier transform analysis of state transitions at 300 K (Fig. S5) indicated that electron–phonon coupling was enhanced by more than 150% under strain^46^, which significantly reduced the electronic excitation barrier through non-adiabatic effects. Moreover, substantial restructuring of the band edge in Al_2_O_3_:Cr^3+^ systems was triggered by a strain of 0–5% along the c-axis, as shown via rigorous band alignment correction^47,48^, resulting in the bending of VBM and CBM by 0.5 eV and 1 eV, respectively (Fig. S6). This strain-mediated hybridization of the band edge significantly enhances the coupling between shallow defect states and band edges^11^, creating additional carrier channels for radiative recombination. Upon stress application, the bound carriers stored in Cr_Al_ activator sites were ionized, transitioning the defect charge state from the ground-state Cr_Al_^0^(^4^A_2_) to the ionized-state Cr_Al_^1+^. Driven by band bending, an enhanced dipole moment, and strengthened electron–phonon coupling, these carriers subsequently underwent relaxation. This process returned the defect charge state to the excited-state Cr_Al_^0^(^2^E), ultimately leading to radiative recombination between electrons and holes at the luminescence center of Cr_Al_ (Fig. 1g).

**Fig. 2:**
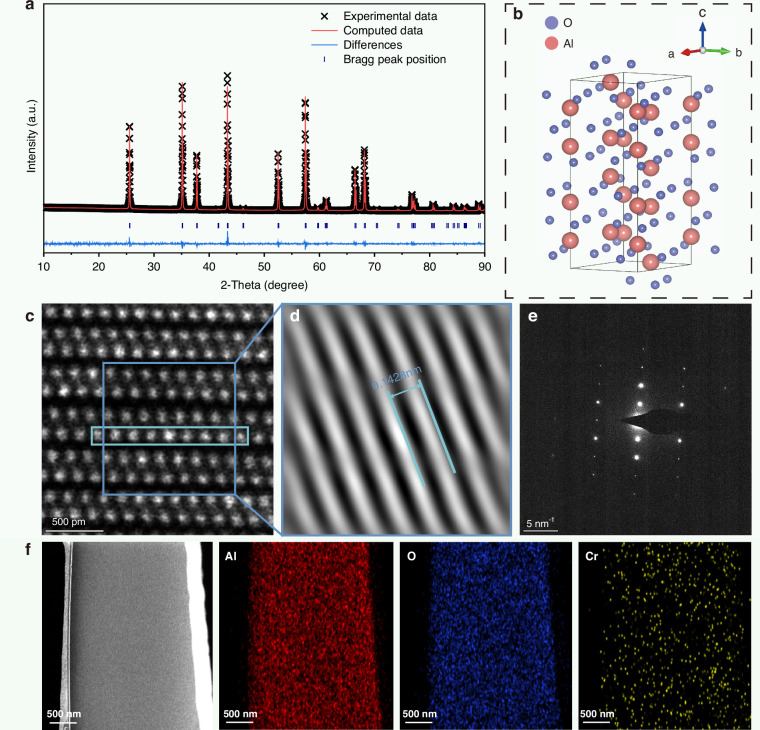
Structure and composition of Al_2_O_3_: Cr^3+^. **a** Rietveld refinement profile of Al_2_O_3_:1%Cr^3+^, confirming the corundum phase with high fitting accuracy. **b** Schematic illustration of the Al_2_O_3_ crystal structure, where Al^3+^ ions occupy octahedral interstices within a hexagonal close-packed oxygen framework. **c** HRTEM image showing the atomic lattice of Al_2_O_3_:1%Cr^3+^. **d** FFT pattern obtained from the dark-framed region in (**c**), revealing distinct lattice fringes and an interplanar spacing of 0.1428 nm. **e** Selected area electron diffraction pattern, confirming the crystalline nature of the sample. **f** Elemental mapping images of Al, O, and Cr from energy-dispersive X-ray spectroscopy, demonstrating a uniform elemental distribution in the Al_2_O_3_:1%Cr^3+^ sample

To investigate the effect of Cr^3+^ doping on the crystal structure of Al_2_O_3_, Rietveld refinements were performed on Al_2_O_3_:1%Cr^3+^ and undoped Al_2_O_3_ to assess their phase purities (Fig. 2a, S7a). The refinement results indicated that both samples exhibited a typical corundum phase structure (space group R-3c, No. 167), with good agreement between the experimental data and fitted curves. The refinement factors were R_wp_ = 5.68% and R_p_ = 4.33% for Al_2_O_3_, and R_wp_ = 6.44% and R_p_ = 4.38% for Al_2_O_3_:1%Cr^3+^. Compared with undoped Al_2_O_3_ (a = b = 4.761 Å, c = 12.997 Å), Al_2_O_3_:1%Cr^3+^ exhibited slightly increased lattice parameters (a = b = 4.763 Å, c = 13.004 Å), which can be attributed to the partial substitution of smaller Al^3+^ ions (0.535 Å) by larger Cr^3+^ ions (0.615 Å). Figure S7b shows a comparison of the X-ray diffraction (XRD) patterns of Al_2_O_3_:1%Cr^3+^ and undoped Al_2_O_3_ with the standard PDF#10-0173. All diffraction peaks matched well with the standard pattern. In addition, Fig. S8a, b show the XRD patterns of Al_2_O_3_:1%Cr^3+^ samples annealed at different temperatures (1573–1923 K) and Al_2_O_3_:xCr^3+^ samples with various doping concentrations (x = 0.01, 0.1, 0.5, 1, 2, and 4%) annealed at 1923 K, respectively. All diffraction peaks of Al_2_O_3_:Cr^3+^ samples were consistent with the standard data for Al_2_O_3_ (PDF#10-0173), indicating that Cr^3+^ was successfully incorporated into the Al_2_O_3_ lattice without altering the host phase structure. A schematic of the Al_2_O_3_ crystal structure is shown in Fig. 2b, where oxygen ions form a hexagonal close-packed array and Al^3+^ ions occupy partial octahedral interstitial sites, forming stable [AlO_6_] coordination environments that provide ideal sites for Cr^3+^ ion substitution. To further verify the crystal structure of Al_2_O_3_:1% Cr^3+^, high-resolution transmission electron microscopy (HRTEM) was conducted on samples prepared via focused ion beam milling. The HRTEM image clearly reveals the atomic arrangement of Al_2_O_3_ (Fig. 2c). The dark-blue region in Fig. 2c was subjected to fast Fourier transform (FFT), and the resulting diffraction pattern clearly exhibited the lattice features of the sample (Fig. 2d). Fig. S9a shows an area scan of the light-blue region in Fig. 2c, revealing a pronounced increase in brightness in the central region, which is attributed to the incorporation of Cr^3+^ ions. With a higher atomic number (Z = 24) than that of Al (Z = 13), Cr exhibits stronger electron scattering. As a result, the incorporation of Cr^3+^ ions into the lattice led to enhanced local contrast in the HRTEM image^49^. This result provides additional evidence of the successful incorporation of Cr^3+^ into the Al_2_O_3_ lattice. Fig. S9b shows the line profile in Fig. 2d, from which the lattice spacing was measured to be 0.1428 nm. A selected area electron diffraction pattern of the sample is shown in Fig. 2e. Figure 2f shows the TEM image and the corresponding energy-dispersive X-ray spectroscopy (EDS) elemental mapping, confirming the presence of Al, O, and Cr in the sample. The elements were uniformly distributed throughout the sample, as observed in the mapping results. Additional scanning electron microscopy (SEM) images and EDS mappings (Fig. S10) further verified the uniform elemental distribution and reliability of the doping process at high Cr^3+^ concentrations (4%).

The effects of different Cr^3+^ doping concentrations and annealing temperatures on the PL and ML properties of Al_2_O_3_:xCr^3+^ were investigated. Fig. S11a shows the PL excitation (PLE) and PL spectra of Al_2_O_3_:xCr^3+^ samples with various Cr^3+^ concentrations (x = 0.01–4%) after annealing at 1923 K. When monitored at an emission wavelength of 694 nm, the PLE spectra exhibited two broad and intense absorption bands centered at 404 and 558 nm, corresponding to the spin-allowed transitions of Cr^3+^ ions: ^4^A_2_(^4^F) → ^4^T_1_(^4^F) and ^4^A_2_(^4^F) → ^4^T_2_(^4^F). A sharp R-line emission at 694 nm was observed in the PL spectra, attributed to the spin-forbidden ^2^E → ^4^A_2_ transition of Cr^3+^ ions in a strong crystal field. This transition is spin-forbidden, and the resulting emission is narrow and exhibits high optical stability. The emission intensity was significantly influenced by the doping concentration, reaching a maximum at x = 0.5%. At lower concentrations (x < 0.5%), the emission intensity was weak due to an insufficient number of luminescent centers. At higher concentrations (x > 0.5%), the reduced distance between Cr^3+^ ions facilitated energy transfer, promoting non-radiative transitions and resulting in a typical concentration quenching effect with significantly reduced emission intensity. Notably, the positions of excitation and emission peaks remained unchanged across all doping concentrations.

Under excitation at 404 nm and monitoring at 694 nm, the fluorescence decay curves of the samples followed a single-exponential fitting function:

1 $$I\left(t\right)={I}_{0}\times {e}^{-\frac{t}{\tau }}$$

where $I\left(t\right)$ represents the fluorescence intensity at time t, ${I}_{0}$ is the initial intensity (at t = 0), and τ represents the fluorescence lifetime. Fig. S11b illustrates the influence of the doping concentration on the PL lifetime. As the Cr^3+^ concentration increased, τ gradually decreased from 3.50 to 0.05 ms. This reduction is attributed to the shorter distances between Cr^3+^ ions, which enhance non-radiative transitions^50^. Fig. S11c shows the PLE and PL spectra of Al_2_O_3_:1%Cr^3+^ samples annealed at temperatures ranging from 1573 to 1923 K. Similarly, the annealing temperature did not affect the positions of excitation and emission peaks. However, the PL intensity increased with increasing annealing temperature, indicating that higher temperatures help optimize the local environment of Cr^3+^ centers and improve radiative recombination efficiency. Fig. S11d shows the variation in the fluorescence lifetime with the annealing temperature. As the annealing temperature increased, τ gradually increased from 0.85 to 2.3 ms. Thermoluminescence (TL) measurements were performed to characterize the trap states in Al_2_O_3_:Cr^3+^ (Fig. S12). A dominant TL peak centered at approximately 546 K indicates the presence of thermally stable traps. The corresponding trap depth, estimated using the improved peak position method, is about 1.41 eV. This indicates a much deeper trap depth compared with that of persistent luminescent phosphors of ~0.5–0.8 eV^33^.

**Fig. 3:**
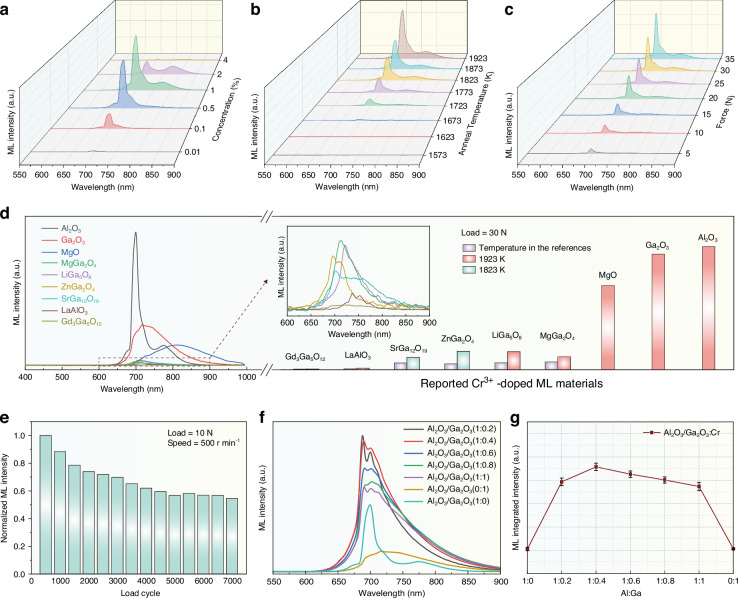
ML properties of Al_2_O_3_:Cr^3+^ and enhancement strategies. **a** ML spectra of Al_2_O_3_:xCr^3+^ samples (x = 0.01, 0.1, 0.5, 1, 2, and 4%) under an applied force of 30 N. **b** ML spectra of Al_2_O_3_:1%Cr^3+^ samples annealed at different temperatures (1573–1923 K) under an applied force of 30 N. **c** ML spectra of Al_2_O_3_:1%Cr^3+^ under varying applied loads ranging from 5 to 35 N. **d** Reported ML spectra of Cr^3+^-doped material systems and comparison of their integrated ML intensities. **e** ML repeatability of Al_2_O_3_:Cr^3+^ under 7000 loading cycles at 10 N without any pre-irradiation. **f** ML spectra of Al_2_O_3_/Ga_2_O_3_:Cr^3+^ heterojunctions with varying Al_2_O_3_ to Ga_2_O_3_ ratios, indicating compositional tuning effects on emission characteristics. **g** Integrated ML intensity of Al_2_O_3_/Ga_2_O_3_:Cr^3+^ heterojunctions with varying Al_2_O_3_ to Ga_2_O_3_ ratios

To evaluate ML performance, the phosphor was embedded in a polyethylene terephthalate (PET) matrix and tested using a custom-built setup. A representative demonstration of ML from the Al_2_O_3_:1%Cr^3+^–PET composite under mechanical scratching is provided in Video S1. Meanwhile, infrared thermal imaging recorded during the scratching process confirms that no discernible surface temperature rise occurs during ML generation, excluding a thermal-origin contribution (Video S2). Figure 3a shows the ML spectra of samples with varying Cr^3+^ doping concentrations under a constant applied force of 30 N. The peak positions and spectral shapes closely matched those of the PL spectra. The maximum ML intensity was observed at a doping concentration of 1% Cr^3+^, whereas higher concentrations led to luminescence quenching^51^. Notably, no detectable ML signal was observed for undoped Al_2_O_3_ under identical testing conditions, further demonstrating that the ML behavior is induced by Cr^3+^ doping rather than the host lattice itself. Figure 3b shows the ML spectra and corresponding intensities of samples annealed at different temperatures, all tested under a constant applied force of 30 N. With increasing annealing temperature, the ML intensity increased significantly, indicating that high-temperature annealing effectively enhances ML performance. Figure S13a, b summarizes the correlations among the integrated ML intensity, doping concentration, and annealing temperature, further confirming the trends discussed above.

The strategies for enhancing the ML performance of Al_2_O_3_:Cr^3+^ were systematically investigated. Fig. S13c shows the effect of varying holding times (1–6 h) on the ML intensity, and Fig. S13d shows the integrated ML intensity of Al_2_O_3_:1%Cr^3+^ at different holding durations. The results indicated that the ML intensity initially increased and then decreased with prolonged holding time, reaching a maximum at 3 h. A further increase to 6 h led to a significant decline in intensity, suggesting the existence of an optimal thermal treatment window. Fig. S13e demonstrates the influence of different annealing atmospheres on ML performance for all samples annealed at 1923 K. Compared to single-atmosphere annealing, the sample treated by a stepwise annealing process—first in air for 2 h followed by an N_2_/H_2_ mixed atmosphere for another 2 h—exhibited the highest ML emission intensity. This enhancement may be attributed to a synergistic optimization mechanism during stepwise atmospheric annealing: the initial air treatment stabilizes the Cr^3+^ luminescent centers, while the subsequent N_2_/H_2_ treatment introduces an appropriate concentration of oxygen vacancies (V_O_), collectively optimizing the carrier trapping and release processes to boost the ML intensity.

To satisfy the practical application requirements, the mechanical response characteristics of Al_2_O_3_:Cr^3+^ were evaluated. Figure 3c illustrates the effects of varying external stress (5–35 N) on the ML intensity and its integrated intensity. The ML intensity increased progressively with increasing applied load, whereas the emission peak position remained unchanged, indicating an excellent mechanoresponsive behavior. Moreover, the integrated intensity exhibited an almost linear increase with the applied load (Fig. S13f). Figure S14 further shows that the NIR ML intensity increases nearly linearly under higher applied loads from 1000 to 4000 N, demonstrating stable luminescence behavior even under large mechanical stress. Variable-temperature ML measurements were carried out from 233 to 373 K under identical mechanical loading conditions to evaluate the thermal robustness of Al_2_O_3_:1%Cr^3+^. Representative ML spectra at different temperatures are shown in Fig. S15a, while the corresponding temperature dependence of the integrated ML intensity is summarized in Fig. S15b. With increasing temperature, the ML intensity exhibits a gradual decrease. This behavior is commonly observed in ML materials and is generally associated with enhanced non-radiative relaxation at higher temperatures. Fig. S16 presents the XRD patterns and ML responses of Al_2_O_3_:1%Cr^3+^ samples after storage in air for one year and after water immersion for one month. No noticeable structural change or degradation of NIR ML intensity is observed, indicating that the material maintains its structural integrity and ML response under these environmental conditions. Fig. S17 shows that the Al_2_O_3_:1%Cr^3+^ exhibits millisecond-scale decay behavior, with an extracted ML lifetime of about 2.1 ms, comparable in timescale to the corresponding PL lifetime. Notably, among reported Cr^3+^-doped materials, Al_2_O_3_:Cr^3+^ exhibited the highest ML performance, as shown in Fig. 3d. The inset shows a magnified view of the dotted line region in the spectrum. The bar chart displays the ML integrated intensity for each material. Based on the above findings regarding the annealing-temperature dependence, the annealing temperature for the other Cr^3+^-doped materials was increased to 1923 K for comparison. Because ZnGa_2_O_4_ and SrGa_12_O_19_ melt at this temperature, these two materials were annealed at 1823 K instead. After high-temperature annealing, a significant enhancement in ML intensity was observed. All samples were synthesized via high-temperature solid-state reactions. The synthesis conditions and corresponding ML intensities are summarized in Table 1, and the XRD patterns and PL spectra are shown in Figs. S18 and S19, respectively. To quantitatively evaluate the self-recoverable nature of the ML response, cyclic loading tests were performed on the Al_2_O_3_:Cr^3+^/PET composite with an applied force of 10 N and a rotation speed of 500 r min⁻¹. As shown in Fig. 3e, although a moderate decrease in intensity occurs during the initial cycles, the ML signal remains stable and repeatedly triggerable over long-term cycling. Even after 7000 loading cycles, approximately 54% of the initial ML intensity is retained.

**Table 1:** Reported Cr^3+^-doped material systems: synthesis conditions and ML intensity

Host	Concentration	Temperature	Time	Peak Intensity (a.u.)	Integrated Intensity (a.u.)	Ref.
Al_2_O_3_	1%	1923 K	4 h	5808 (P = 694 nm)	220,728	This work
Ga_2_O_3_	1%	1923 K	4 h	1768 (P = 716 nm)	207,100	^35^
MgO	0.5%	1923 K	4 h	883 (P = 808 nm)	151,137	^39^
MgGa_2_O_4_	0.5%	1623 K	6 h	206 (P = 712 nm)	14,064	^36^
LiGa_5_O_8_	0.6%	1673 K	6 h	192 (P = 721 nm)	12,551	^33^
ZnGa_2_O_4_	0.5%	1673 K	6 h	160 (P = 696 nm)	10,687	^34^
SrGa_12_O_19_	0.5%	1733 K	6 h	115 (P = 702 nm)	12,228	^34^
LaAlO_3_	0.6%	1773 K	10 h	48 (P = 737 nm)	1844	^40^
Gd_3_Ga_5_O_12_	3%	1673 K	6 h	13 (P = 719 nm)	1366	^37^

Previous studies have shown that the presence of anomalous bonding states at heterojunction interfaces can induce significant band offsets, lowering the energy barrier for electron transitions associated with ML and thereby enhancing ML performance^52^. Inspired by this, this work synthesized Al_2_O_3_/Ga_2_O_3_:Cr^3+^ heterojunction materials by introducing Ga_2_O_3_ and adjusting the phase ratio to optimize ML behavior. As shown in Fig. S20, the XRD patterns of Al_2_O_3_/Ga_2_O_3_:Cr^3+^ samples with different ratios confirmed the presence of both Ga_2_O_3_ (PDF#76-0573) and Al_2_O_3_ (PDF#10-0173) phases, indicating the successful formation of hybrid heterostructures. The PL properties of heterojunction samples were further investigated (Table S1). With increasing Ga_2_O_3_ content, the excitation peaks (Ex I and Ex II) red-shifted from 402 nm and 558 nm to 424 nm and 589 nm, corresponding to the Cr^3+^ transitions of ^4^A_2_(^4^F) → ^4^T_1_(^4^F) and ^4^A_2_(^4^F) → ^4^T_2_(^4^F), respectively. Furthermore, the emission peak Em II shifted from 694 nm to 702 nm, accompanied by the emergence of two additional peaks, Em I and Em III. Em I/Em II were attributed to the narrow-line ^2^E → ^4^A_2_ transitions (R1 and R2 lines), while Em III corresponded to the broad ^4^T_2_ → ^4^A_2_ transition. In addition, the full width at half maximum of the PL spectrum markedly increased from 2 nm to 92 nm, which may have resulted from new energy-level structures or local electronic environment changes induced at the heterojunction interface. Figure 3f shows the ML properties of Al_2_O_3_/Ga_2_O_3_:Cr^3+^ samples with varying heterojunction ratios. As the Ga_2_O_3_ content increased, the ML spectra exhibited red-shifting and band-broadening behavior similar to that observed in the PL spectra, which was attributed to the weakened crystal field strength from the higher Ga^3+^ content. Figure 3g shows the integrated ML intensity as a function of the Al_2_O_3_/Ga_2_O_3_ ratio, with the highest value observed at a ratio of 1:0.4. This indicates that, at this composition, the interface region effectively facilitates charge carrier migration and recombination, leading to optimal ML enhancement.

Table S2 summarizes the quantum yield (QY) performances of Al_2_O_3_:Cr^3+^ and Al_2_O_3_/Ga_2_O_3_:Cr^3+^ heterojunction materials under different Cr^3+^ doping concentrations and annealing temperatures. The QY of Al_2_O_3_:Cr^3+^ decreased markedly with increasing Cr^3+^ concentration but improved significantly with higher annealing temperatures. In Al_2_O_3_/Ga_2_O_3_:Cr^3+^ heterojunctions, the QY slightly decreased with increasing Ga_2_O_3_ content; however, optimized Cr^3+^ doping still delivered near-ideal efficiency for NIR emissions.

**Fig. 4:**
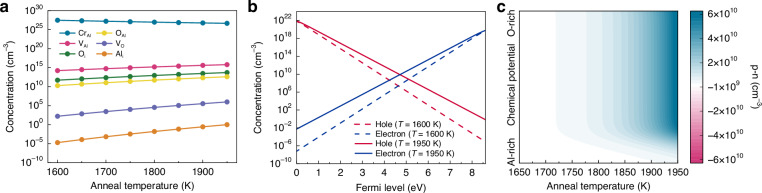
DFT explanation of the temperature-dependent ML properties of Al_2_O_3_:Cr^3+^. **a** Equilibrium defect concentration at 300 K in Al_2_O_3_ crystals grown at 1950 K as a function of the annealing temperature. **b** Carrier concentration at the initial annealing temperature of 1600 K and the optimal annealing temperature of 1950 K. **c** Calculated carrier concentration in Al_2_O_3_:Cr^3+^ at room temperature, as a function of annealing temperature and chemical potential conditions

Furthermore, this work performed DFT calculations to gain insights into the influence of temperature on ML performance. Our results revealed that the thermally activated exponential increase in the defect concentration (Fig. 4a) underlay the luminescence enhancement observed at elevated annealing temperatures, aligning with experimental observations. The relationship between the carrier concentration and the self-consistent Fermi level (E_F_) further demonstrated an increase by an order of magnitude as the temperature increased from 1600 K to 1950 K (Fig. 4b). This exponential increase in carrier concentration with annealing temperature was consistently observed under various chemical synthesis conditions (Fig. 4c). The resulting higher carrier density significantly enhanced the radiative recombination efficiency, leading to superior luminescence performance.

**Fig. 5:**
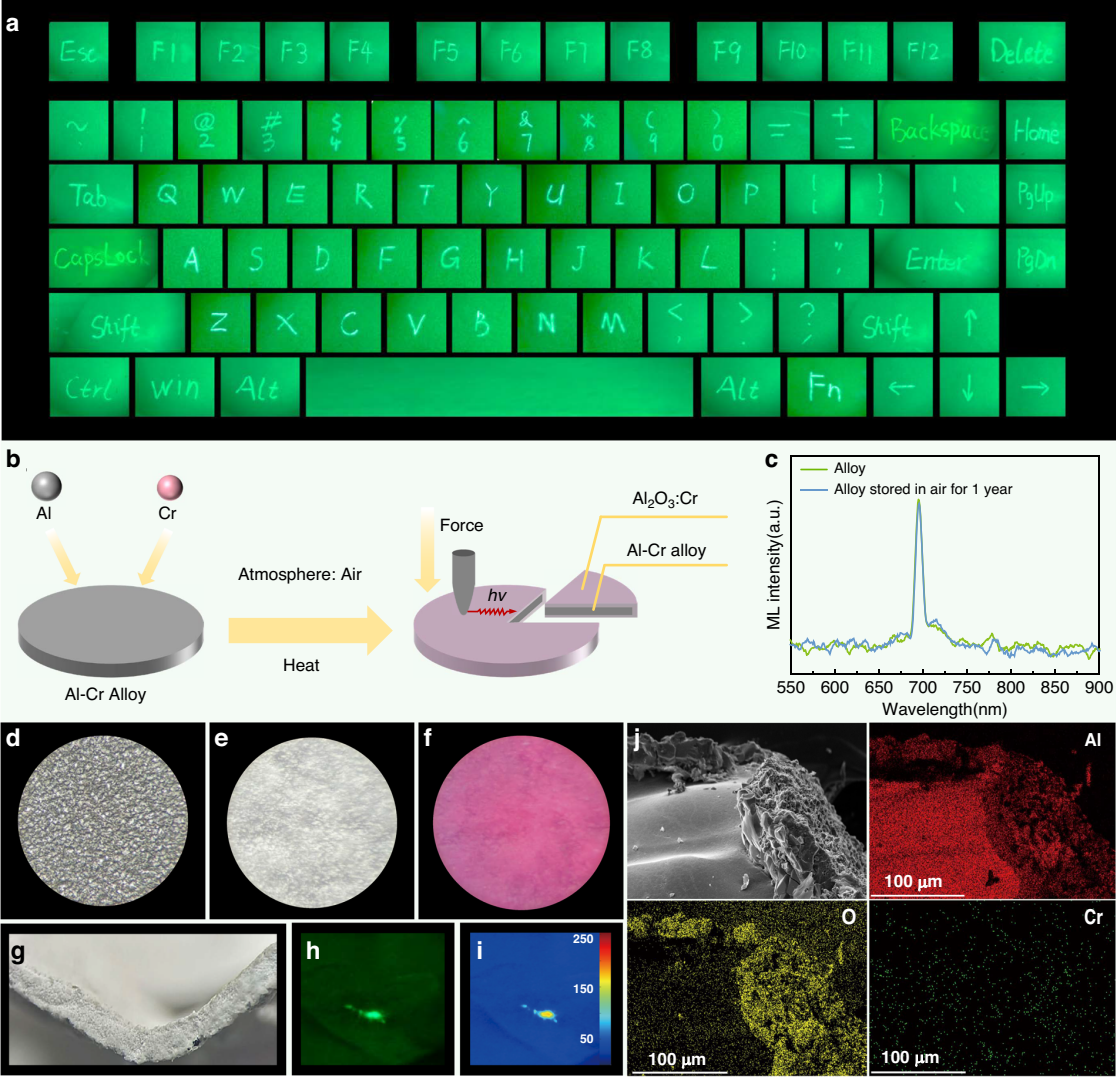
Application exploration of Al_2_O_3_:Cr^3+^ **a** Visualization of a handwritten keyboard pattern on the ML paper. **b** Schematic illustration of the formation of an Al_2_O_3_:Cr^3+^ thin film on the Cr–Al alloy via thermal oxidation. **c** ML emission of the Cr–Al alloy after 1-year natural storage under ambient conditions. Surface morphology of the alloy (**d**) before thermal treatment and (**e**) after thermal oxidation. **f** PL of the Al_2_O_3_:Cr^3+^ thin film under 365-nm UV excitation. **g** Flexibility of the Cr–Al alloy with the Al_2_O_3_:Cr^3+^ thin film under bending deformation. **h** ML image of the Al_2_O_3_:Cr^3+^ thin film under external force application, and (**i**) the corresponding grayscale intensity map. **j** Cross-sectional SEM image and EDS elemental mapping of the Cr–Al alloy and the surface of the Al_2_O_3_:Cr^3+^ thin film

The following section describes the application of this simple oxide system on both flexible and rigid substrates. For flexible substrates, a novel ML paper was designed and fabricated using Al_2_O_3_/Ga_2_O_3_:Cr^3+^ heterojunction materials, leveraging their high emission brightness, excellent thermal stability, and environmental durability. Potential applications in anti-counterfeiting and stress visualization were explored. The ML paper was successfully fabricated by mixing Al_2_O_3_/Ga_2_O_3_:Cr^3+^ powder with dehydrated paper pulp at a mass ratio of 4:1, followed by a conventional handmade papermaking process, yielding a material with outstanding mechano-optical responsiveness. Under visible light, the paper retained its natural white appearance; however, under UV illumination at 365 nm, it emitted distinct NIR fluorescence (Fig. S21a). Notably, the embedded powder retained its excellent ML performance, emitting NIR light stably even after incorporation into the paper matrix. Videos S3 and S4 further demonstrate the luminescent behavior of the ML paper under binocular night-vision observation. Distinct ML signals were observed when scratching with a glass rod or cutting with scissors, highlighting the unique stress-triggered emission pattern. This emission pattern is difficult to replicate, enhancing the technical reliability of multi-level anti-counterfeiting and invisible data encryption, thereby offering robust support for applications such as secure labeling and personalized information storage. To evaluate the long-term environmental stability of the ML paper, samples were stored under ambient laboratory conditions for up to one year. After storage, the ML paper still exhibited excellent ML emission under mechanical stimulation, with no obvious degradation compared to its initial state (Fig. S21b). Furthermore, the mechanical fatigue stability of the ML paper was examined through repeated loading–unloading tests. As shown in Fig. S21c, the ML intensity remained at a relatively stable level over 100 consecutive mechanical cycles, indicating good durability under repeated mechanical stimulation. In a darkroom environment, motion trajectories written on the paper were clearly visualized using binocular night-vision devices. Figure 5a shows a handwritten keyboard pattern on the ML paper, clearly capturing the motion of the applied forces. Additionally, Fig. S21d shows the handwritten emission trails of numbers 1 through 9 and letters a through z, further demonstrating the potential of this material for stress visualization applications. In addition, a composite polydimethylsiloxane (PDMS) film was fabricated by mixing Al_2_O_3_:Cr^3+^, ZnS:Cu^+^, and PDMS in a mass ratio of 1:1:2, which exhibited both visible and NIR PL and ML emissions (Fig. S22a, b). Video S5 shows the stretching-induced luminescence of the PDMS film recorded using both infrared and visible cameras with a 600-nm high-pass filter. In addition, the ML response of the Al_2_O_3_:Cr^3+^/PDMS composite can also be triggered under non-contact ultrasonic excitation, as demonstrated in Video S6.

In addition to its application on flexible substrates, this material system demonstrates promising potential for stress visualization on rigid substrates, which are widely used in practical engineering scenarios. Aluminum alloys are widely employed as structural materials in the aerospace, automotive, and other industries owing to their low density and high strength. However, in practical applications, structural components often experience complex and nonuniform stress distributions, which vary significantly with the working conditions. Although finite element simulations are typically employed during the design phase to predict stress distributions and optimize structures, discrepancies often exist between the simulated results and actual stress states in real-world scenarios. To address this issue, we proposed a novel stress-responsive optical monitoring strategy involving the in-situ construction of an Al_2_O_3_:Cr^3+^ ML layer on Cr–Al alloy surfaces via thermal oxidation, enabling passive and visual monitoring of ML. A schematic illustration of the thermally induced formation of the Al_2_O_3_:Cr^3+^ layer on Cr–Al alloys is shown in Fig. 5b. Upon heating the Cr–Al alloy in air, surface oxidation occurred, leading to the formation of a uniform Al_2_O_3_:Cr^3+^ layer. The in situ-formed layer exhibited ML upon mechanical stimulation. After long-term natural storage under ambient conditions for up to one year, the in situ-formed Al_2_O_3_:Cr^3+^ layer on the Cr–Al alloy still exhibited clear ML emission under mechanical stimulation, demonstrating good environmental stability (Fig. 5c).

The XRD pattern of the Cr–Al alloy is shown in Fig. S23a. The alloy surface exhibited a metallic luster before heating (Fig. 5d), whereas it became rough and oxidized after heating (Fig. 5e). Under 365-nm UV excitation, the oxide layer displayed distinct PL (Fig. 5f) while maintaining sufficient flexibility (Fig. 5g) to accommodate various complex deformations encountered in practical applications. Furthermore, mechanical fatigue stability was evaluated by subjecting the Cr–Al alloy to repeated mechanical loading–unloading cycles. As shown in Fig. S23b, the ML intensity remained relatively stable over 100 consecutive cycles, indicating good durability of the oxide layer under repeated mechanical stimuli. Fig. S24a shows the PL spectrum of the Al_2_O_3_:Cr^3+^ layer under 402-nm excitation, showing a sharp emission peak at 694 nm. The fluorescence decay curves at 402-nm and 558-nm excitation, monitored at 694 nm, exhibited millisecond-scale lifetimes (Fig. S24b). The temperature-dependent PL spectra (Fig. S24c, d) showed a gradual intensity variation with increasing temperature, whereas the emission peak position remained nearly unchanged. Upon external mechanical loading, the layer emitted stable NIR ML (Fig. 5h and Video S7). The color-encoded grayscale image (Fig. 5i) clearly reveals the correlation between the luminescence intensity and applied stress, demonstrating the excellent pressure sensitivity of the material. Cross-sectional SEM and EDS elemental mapping (Fig. 5j) further confirmed the compact structure and uniform elemental distribution of the oxide layer. Al was distributed throughout the sample, originating from the alloy substrate and extending into the oxide layer. Oxygen was primarily enriched at the surface, reflecting the oxidation process, whereas chromium was uniformly distributed within the layer, indicating its successful incorporation as a luminescent center.

Compared with conventional stress-sensing technologies, this Al_2_O_3_:Cr^3+^-based method does not require an external power supply or complex electronics and relies solely on stress-induced optical signals for real-time stress detection and visualization. The in-situ growth process ensures robust interfacial bonding between the oxide layer and the alloy substrate. The resulting dense and uniform layer exhibits excellent mechanical stability and flexibility, making it suitable for structural monitoring in complex environments. This method provides a novel optical solution for dynamic structural status detection and health monitoring and shows significant promise for high-end engineering applications, particularly in the aerospace, energy pipelines, and large-scale industries. This straightforward thermal treatment enables the functionalization of structural materials, thereby laying a solid foundation for the development of next-generation smart components with self-sensing capabilities.

## Discussion

In summary, this work demonstrated that Al_2_O_3_:Cr^3+^ (in the form of crystals, powders, and Al alloy-related products) is a robust and scalable oxide platform for self-recoverable NIR ML. DFT calculations confirmed that this material exhibited efficient self-recovery behavior combined with a high-brightness emission potential. Guided by these mechanistic insights, we optimized the material via controlled doping, high-temperature annealing, and Ga_2_O_3_ heterostructure engineering, thereby significantly boosting ML efficiency and stability. Validated across both flexible and rigid devices, the material enables applications such as NIR ML stress-mapping and anti-counterfeiting paper, as well as real-time structural monitoring of layers formed on Cr–Al alloys. This work clarifies the mechanical-to-optical conversion mechanism, bridges fundamental photonic research with practical stress-sensing technologies, and provides a general strategy for designing oxide-based ML systems for intelligent photonic and sensing applications.

## Materials and methods

### Materials

Al_2_O_3_ (99.99%, Sinopharm Co., Ltd.), Ga_2_O_3_ (99.99%, Aladdin), and Cr_2_O_3_ (99.95%, Aladdin) were used as starting materials.

### Preparation method

Al_2_O_3_: xCr^3+^ samples (x = 0.01, 0.1, 0.5, 1, 2, and 4%) were prepared using the high-temperature solid-phase method. First, Al_2_O_3_ and Cr_2_O_3_ powders were accurately weighed according to the desired Cr^3+^ doping ratio. The raw materials were wet-ground in an agate mortar with a small amount of anhydrous ethanol for 40 min. The resulting slurry was dried at 80 °C, placed in a corundum crucible, and compacted. The mixtures were annealed in a chamber furnace at different temperatures (1573, 1623, 1673, 1723, 1773, 1823, 1873, and 1923 K) for 4 h in air. After sintering, the samples were cooled naturally to room temperature and sieved through a 150-mesh sieve to obtain a homogeneous powder for subsequent testing and characterization.

Al_2_O_3_/mGa_2_O_3_:1% Cr^3+^ samples (m = 0.2, 0.4, 0.6, 0.8, and 1) were prepared using the high-temperature solid-phase method. Al_2_O_3_, Ga_2_O_3_, and Cr_2_O_3_ powders were first weighed in stoichiometric ratios and subjected to the same mixing, wet milling, and drying procedures described above. The treated powders were then placed in a corundum crucible, compacted, and annealed in a box furnace at 1923 K for 4 h to complete the reaction in air. After sintering, the samples were naturally cooled to room temperature and sieved through a 150-mesh sieve to obtain a homogeneous powder for subsequent testing and characterization.

### Preparation method for ML test films

For ML films, 0.3 g of the powder and 0.06 g of UV curing adhesive (LEAFTOP 9307) were added to a test tube along with 9 mL of anhydrous ethanol. The mixture was shaken well and placed in an ultrasonic cleaner for ultrasonic dispersion to ensure uniformity. The well-dispersed mixture was poured into a 3 × 3-cm square mold and left to stand until the ethanol completely evaporated. After film formation, the mold was removed, and the dried powder layer was sandwiched between the top and bottom layers of the Ethylene Vinyl Acetate (EVA)–PET plastic sealing film (Deli No. 3817). The assembly was then irradiated and cured using a UV lamp to ensure that the layers were firmly bonded. Finally, the sandwich film was placed in a hot-press laminator for pressing and laminating to enhance its mechanical strength and ensure uniform thickness, yielding a highly stable ML test film for subsequent mechanical testing.

### Material characterization

XRD measurements were performed using a SmartLab multifunctional diffractometer (Rigaku, Japan) operated at 45 kV and 200 mA. ML measurements were performed using a custom-built testing system comprising a digital force gauge, uniaxial controller, stepper motor, fiber-optic spectrometer (Ocean Optics QE65Pro), glass substrate, and a computer. For testing, the ML film/paper was fixed on a glass plate, and a metal probe with a hemispherical tip of 0.1 mm was brought into contact with the film by adjusting the force gauge position to apply pressure. The opposite side of the glass plate was connected to an optical fiber coupled to a spectrometer for light collection. The uniaxial controller drove the stepper motor to move the slider and fiber assembly horizontally, enabling real-time acquisition of dynamic ML signals. For the cyclic repeatability test, the ML powder was first encapsulated in a PET film. Cyclic mechanical stimulation was applied using a motor-driven setup with a rotation speed of 500 r min⁻¹ under a constant normal force of 10 N. The emitted ML signal during repeated cycles was collected by an optical fiber and recorded using a spectrometer. For large-pressure ML measurements, the ML powder was embedded in an epoxy resin matrix with a mass ratio of powder to resin of 1:4. After curing, the composite sample was subjected to external mechanical loading. For the Cr–Al alloy sample, ML measurements were carried out by scraping the alloy surface under an applied force of 3 N. An optical fiber was covered with a glass test tube and positioned on the same side of the scraping region to collect the emitted ML signal, which was then recorded by a spectrometer. PL, fluorescence lifetime, and PLQY were characterized using an FLS1000 spectrofluorometer (Edinburgh Instruments Ltd., UK) equipped with a xenon lamp as the excitation source. Temperature-dependent PL measurements were performed using a Hitachi F-7100 spectrofluorometer (Japan) with continuous excitation from a 405-nm laser for 16 min. A 510-nm optical filter was used to eliminate residual excitation light. The sample temperature was regulated and recorded using a heating stage combined with an infrared thermal imager. Spectral data were collected using an Ocean Optics USB65 Pro spectrometer at an integration time of 100 ms and stored at 10-°C intervals. SEM images were obtained using a Thermo Scientific APREO S high-resolution field-emission scanning instrument, whereas TEM images were acquired using a Thermo Fisher Titan Cubed Themis G2 300 microscope equipped with a double spherical aberration correction.

### Computational methodology

This study employed first-principles calculations based on density functional theory (DFT), as implemented in the Vienna Ab initio Simulation Package (VASP). The electron–ion interactions were described using the projector augmented-wave (PAW) pseudopotential method, with a plane-wave cutoff energy of 520 eV. Structural optimizations were performed using the PBEsol functional, and Brillouin zone integrations were carried out on a 3 × 3 × 3 k-point mesh. To accurately capture the localized nature of Cr 3 d orbitals, electronic structure calculations were conducted using the HSE06 hybrid functional. In these calculations, the screened Fock exchange mixing parameter *α* was set to 0.31, a value optimized to reproduce the experimental band gap of Al_2_O_3_ (DFT 8.61 eV, Exp. 8.7 eV). This hybrid functional approach effectively mitigates self-interaction error and significantly improves the accuracy of defect energy level predictions. Detailed computational methodology are reported in Supplementary Information.

## Supplementary information

Supplementary Information

Video S1. PET

Video S2. Thermal imaging

Video S3. Paper Bending and Sliding

Video S4. Pepper Cutting

Video S5.PDMS

Video S6. Ultrasound

Video S7. Alloy

## References

[1] Wang, H. et al. Oscillatory mechanoluminescence of Mn^2+^-doped SrZnOS in dynamic response to rapid compression. *Nat. Commun.* **16**, 548 (2025). 39789031 10.1038/s41467-025-55922-x PMC11718179

[2] Jeong, H. I. et al. Super elastic and negative triboelectric polymer matrix for high performance mechanoluminescent platforms. *Nat. Commun.* **16**, 854 (2025). 39833144 10.1038/s41467-025-56007-5 PMC11747495

[3] Cai, C. Y. et al. Multi-stimulated far-UVC luminescence for solar-blind imaging. *Nat. Commun.* **16**, 6224 (2025). 40617860 10.1038/s41467-025-61522-6 PMC12228785

[4] Wang, W. X. et al. Contact-separation-induced self-recoverable mechanoluminescence of CaF_2_:Tb^3+^/PDMS elastomer. *Nat. Commun.* **15**, 2014 (2024). 38443411 10.1038/s41467-024-46432-3 PMC10914845

[5] Chang, K. et al. Achieving ultrasound-excited emission with organic mechanoluminescent materials. *Adv. Mater.* **36**, 2407875 (2024). 10.1002/adma.202407875 39049679

[6] Runowski, M. et al. Sound, force and light induced emissions from Er^3+^-Mn^2+^ doped ZnS/CaZnOS heterostructure for remote temperature monitoring via photo- and mechanoluminescence. *Adv. Mater.* **37**, e10117 (2025). 40696957 10.1002/adma.202510117 PMC12531744

[7] Tao, L. et al. High-quality perovskite quantum dots with excellent reproducibility and amplified spontaneous emission by optimization of cesium precursor. *Light Adv. Manuf.* **6**, 12 (2025).

[8] Lv, X. L. et al. Self-powered mechanoluminescent elastomer for solar-blind ultraviolet emission. *Light Sci. Appl.* **15**, 61 (2026). 41521172 10.1038/s41377-025-02131-2 PMC12791142

[9] Xie, Z. Dual-channel mechano-phosphorescence: a combined locking effect with twisted molecular structures and robust interactions. *Light Sci. Appl.* **13**, 85 (2024). 38589343 10.1038/s41377-024-01421-5 PMC11001961

[10] Sun, X. L. et al. 3D printing of auxetic self-powered mechanoluminescent photonic skins for underwater communication and safety monitoring. *Adv. Mater.* **37**, 2502743 (2025). 40376866 10.1002/adma.202502743 PMC12369689

[11] Wang, X. D. et al. Dynamic pressure mapping of personalized handwriting by a flexible sensor matrix based on the mechanoluminescence process. *Adv. Mater.* **27**, 2324–2331 (2015). 25711141 10.1002/adma.201405826

[12] Guo, J. X. et al. In-sensor computing with visual-tactile perception enabled by mechano-optical artificial synapse. *Adv. Mater.* **37**, 2419405 (2025). 10.1002/adma.202419405 39998263

[13] Liu, S. Q. et al. Bright chromium-sensitized lanthanide NIR-II mechanoluminescence in a piezoelectric oxide. *Adv. Mater.* **37**, e06957 (2025). 40801223 10.1002/adma.202506957

[14] Zhang, P. et al. High defect tolerance breaking the design limitation of full-spectrum multimodal luminescence materials. *Adv. Mater.* **37**, 2411532 (2025). 10.1002/adma.202411532 39668470

[15] Wang, Y. Z. et al. Synthesis of SrZnOSe crystals with low phonon energy for enhancing near-infrared mechanoluminescence. *Adv. Mater.* **36**, 2406899 (2024). 10.1002/adma.202406899 39530652

[16] Liu, S. Q. et al. Chromium-activated phosphors: from theory to applications. *Chem. Soc. Rev.* **55**, 1954–1998 (2026). 41411014 10.1039/d5cs00957j

[17] Dubey, V. et al. Mechanoluminescence behavior of rare-earth-activated phosphors. in Rare-Earth-Activated Phosphors (eds Dubey, V. et al.) (Amsterdam: Elsevier, 2022), 283-319.

[18] Xu, C. N. et al. Direct view of stress distribution in solid by mechanoluminescence. *Appl. Phys. Lett.* **74**, 2414–2416 (1999).

[19] Xu, C. N. et al. Artificial skin to sense mechanical stress by visible light emission. *Appl. Phys. Lett.* **74**, 1236–1238 (1999).

[20] Hou, B. et al. An interactive mouthguard based on mechanoluminescence-powered optical fibre sensors for bite-controlled device operation. *Nat. Electron.* **5**, 682–693 (2022).

[21] Jeong, H. I. et al. Defect localized mechanoluminescence model in copper doped zinc sulfide. *ACS Nano* **19**, 35027–35036 (2025). 41001895 10.1021/acsnano.5c11956

[22] Jeong, H. I. et al. High-resolution mechanoluminescent haptic sensor via dual-functional chromatic filtration by a conjugated polymer shell. *Adv. Mater.* **37**, e08917 (2025). 40810698 10.1002/adma.202508917 PMC12592900

[23] Jeong, H. I. et al. Interfacial dipole moment engineering in self-recoverable mechanoluminescent platform. *Mater. Today* **81**, 4–11 (2024).

[24] Zhang, J. C. et al. An intense elastico-mechanoluminescence material CaZnOS:Mn^2+^ for sensing and imaging multiple mechanical stresses. *Opt. Express* **21**, 12976–12986 (2013). 23736551 10.1364/OE.21.012976

[25] Botterman, J. et al. Mechanoluminescence in BaSi_2_O_2_N_2_:Eu. *Acta Mater.* **60**, 5494–5500 (2012).

[26] Li, H. M. et al. Force-induced ultraviolet C luminescence of Pr^3+^-Doped Sr_2_P_2_O_7_ for X-ray dosimetry. *Adv. Mater.* **36**, 2411804 (2024). 10.1002/adma.202411804 39436098

[27] Ning, J. J. et al. MgF_2_:Mn^2+^: novel material with mechanically-induced luminescence. *Sci. Bull.* **67**, 707–715 (2022). 10.1016/j.scib.2021.12.005 36546135

[28] Zheng, T. et al. Mechanoluminescent aluminum nitride crystal for super-sensitive optical manometry, thermometry and force sensing. *Adv. Mater.* **38**, e11943 (2026). 40923469 10.1002/adma.202511943 PMC12759223

[29] Xiong, P. X. & Peng, M. Y. Near infrared mechanoluminescence from the Nd^3+^ doped perovskite LiNbO_3_:Nd^3+^ for stress sensors. *J. Mater. Chem. C.* **7**, 6301–6307 (2019).

[30] Tiwari, R. et al. Fracto-mechanoluminescence induced by impulsive deformation of II–VI semiconductors. *Luminescence* **30**, 883–890 (2015). 25669489 10.1002/bio.2837

[31] Ming, J. et al. High-brightness transition metal-sensitized lanthanide near-infrared luminescent nanoparticles. *Nat. Photonics* **18**, 1254–1262 (2024). 10.1038/s41596-025-01245-6 40908364

[32] Liu, S. Q. et al. Intervalence charge transfer of Cr^3+^-Cr^3+^ aggregation for NIR-II luminescence. *Light Sci. Appl.* **12**, 181 (2023). 37488126 10.1038/s41377-023-01219-x PMC10366090

[33] Xiong, P. X. et al. Self-recoverable mechanically induced instant luminescence from Cr^3+^-doped LiGa_5_O_8_. *Adv. Funct. Mater.* **31**, 2010685 (2021).

[34] Liu, S. Q. et al. Near-infrared mechanoluminescence of Cr^3+^ doped gallate spinel and magnetoplumbite smart materials. *Adv. Funct. Mater.* **33**, 2209275 (2023).

[35] Suo, H. et al. A broadband near-infrared nanoemitter powered by mechanical action. *Matter* **6**, 2935–2949 (2023).

[36] Yu, X. et al. Intense NIR mechanoluminescence from Al^3+^-regulated MgGa_2_O_4_:Cr^3+^. *Chem. Eng. J.* **491**, 152155 (2024).

[37] Bu, W. F. et al. Near-infrared mechanoluminescence of Gd_3_Ga_5_O_12_:Cr^3+^, La^3+^ for biological stress imaging. *Laser Photonics Rev.* **19**, 2400893 (2025).

[38] Wu, S. et al. Self-powered near-infrared mechanoluminescence through MgO/MgF_2_ piezo-photonic heterojunctions. *Nat. Commun.* **16**, 8912 (2025). 41057357 10.1038/s41467-025-63980-4 PMC12504433

[39] Zhao, F. Y. et al. Self-recoverable broadband near-infrared mechanoluminescence in Cr^3+^-doped MgO. *Sci. China Mater.* **68**, 4440–4447 (2025).

[40] Shao, P. S. et al. Self-recoverable NIR mechanoluminescence from Cr^3+^ doped perovskite type aluminate. *Adv. Powder Mater.* **3**, 100165 (2024).

[41] Mashiko, H. et al. Multi-petahertz electron interference in Cr:Al_2_O_3_ solid-state material. *Nat. Commun.* **9**, 1468 (2018). 29670122 10.1038/s41467-018-03885-7 PMC5906618

[42] Dang, P. P. et al. Recent advances in chromium-doped near-infrared luminescent materials: fundamentals, optimization strategies, and applications. *Adv. Opt. Mater.* **11**, 2201739 (2023).

[43] Zheng, G. J. et al. Rare-metal-free ultrabroadband near-infrared phosphors. *Adv. Mater.* **37**, 2415791 (2025). 10.1002/adma.202415791 39618013

[44] Shang, L. B. et al. Understanding near-infrared luminescence in Cr-doped La-gallogermanates through first-principles calculations and ligand-field theory. *Phys. Rev. B* **108**, 155136 (2023).

[45] Liu, J. et al. An efficient descriptor for rapid determination of dipole moments and band alignments of 2D janus transition-metal dichalcogenides. *Adv. Funct. Mater.* **34**, 2401737 (2024).

[46] Dodani, S. C. et al. Discovery of a regioselectivity switch in nitrating P450s guided by molecular dynamics simulations and Markov models. *Nat. Chem.* **8**, 419–425 (2016). 27102675 10.1038/nchem.2474 PMC4843824

[47] Zhang, Y. Y. et al. Origin of the type-II band offset between rutile and anatase titanium dioxide: classical and quantum-mechanical interactions between O ions. *Phys. Rev. B* **95**, 155308 (2017).

[48] Zhang, Y. et al. Synthesis of mixed-dimensional 1D-graphene nanoribbon/2D-CuSe heterostructures with controllable band alignments. *Nat. Commun.* **16**, 5988 (2025). 40593821 10.1038/s41467-025-60916-w PMC12219289

[49] Borshon, I. Z. et al. Predicting column heights and elemental composition in experimental transmission electron microscopy images of high-entropy oxides using deep learning. *npj Comput. Mater.* **10**, 275 (2024).

[50] Huang, D. C. et al. A highly efficient and thermally stable broadband Cr^3+^-activated double borate phosphor for near-infrared light-emitting diodes. *J. Mater. Chem. C.* **9**, 164–172 (2021).

[51] Zhang, Y. et al. Blue LED-pumped intense short-wave infrared luminescence based on Cr^3+^-Yb^3+^-co-doped phosphors. *Light Sci. Appl.* **11**, 136 (2022). 35562360 10.1038/s41377-022-00816-6 PMC9106724

[52] Peng, D. F. et al. A ZnS/CaZnOS heterojunction for efficient mechanical-to-optical energy conversion by conduction band offset. *Adv. Mater.* **32**, 1907747 (2020). 10.1002/adma.201907747 32128925

