# Supplementary material for: Self-recoverable mechanoluminescence in simple oxides: Al2O3:Cr
Source: Light Sci Appl. 2026 Apr 15;15:200. doi: 10.1038/s41377-026-02274-w (PMC13083877; doi:10.1038/s41377-026-02274-w)
Supplement: Supplementary file 1 — Supplementary Information [file 41377_2026_2274_MOESM1_ESM.docx]

Supplementary Information

Self-Recoverable Mechanoluminescence in Simple Oxides: Al_2_O_3_:Cr

*Ziyi Fang^1^, Xiaofeng Pan^2^, Qi’an Zhang^1^, Mingzhi Wu^1^, Yang Liu^1^, Qidong Ma^1^, Biyun Ren^1^, Yanze Wang^1^, Shengqiang Liu^1^, Maryam Zulfiqar^1^, Ming-Gang Ju^2^*, Jiulin Gan^3^*, Leipeng Li^4^, Feng Wang^5,6^*, and Dengfeng Peng^1,7,8^**

^1^ *College of Physics and Optoelectronic Engineering, Shenzhen University, Shenzhen 518060, China*

^2^ *Key Laboratory of Quantum Materials and Devices of Ministry of Education, School of Physics, Southeast University, Nanjing 21189, China*

^3^ *State Key Laboratory of Luminescent Material and Devices, and Guangdong Provincial Key Laboratory of Fibre Laser Materials and Applied Techniques, Guangdong Engineering Technology Research and Development Center of Special Optical Fiber Materials and Devices, South China University of Technology, Guangzhou, 510641, China*

^4^ *College of Physics Science and Technology, Hebei University, Baoding, China*

^5^ *Department of Materials Science and Engineering, City University of Hong Kong, Hong Kong SAR, China*

^6^ *Hong Kong Institute for Clean Energy, City University of Hong Kong, Hong Kong SAR, China*

^7^ *Key Laboratory of Optoelectronic Devices and Systems of Ministry of Education and Guangdong Province,Shenzhen University, Shenzhen,Guangdong 518060, China*

^8^ *China State Key Laboratory of Radio Frequency Heterogeneous Integration, Shenzhen University, Shenzhen, Guangdong 518060, China*

*These authors contributed equally: Ziyi Fang, Xiaofeng Pan and Qi’an Zhang.*

^*^Corresponding author.

*E-mail:* pengdengfeng@szu.edu.cn (D. Peng)

*E-mail:* juming@seu.edu.cn (M. Ju)

*E-mail*: [msgan@scut.edu.cn](mailto:msgan@scut.edu.cn)(J. Gan)

*E-mail*: [fwang24@cityu.edu.hk](mailto:fwang24@cityu.edu.hk)(F. Wang)

Materials and methods

Computational Methodology

Considering that Al_2_O_3_ is a nonpolar semiconductor with a trigonal crystal structure^1^, uniaxial strains with magnitudes in the range of 0–5% were applied in the DFT calculations^2–4^. In particular, a uniaxial compressive strain of 5% was imposed along the crystallographic *c*-axis. In addition, uniaxial strains were independently applied along the *c*-axis and the *a*-axis (perpendicular to the *c*-axis) to examine the directional dependence of Al_2_O_3_ under mechanical deformation. Compared with the unstrained case, the effects of strain applied along either the *a*-axis or the *c*-axis on the electronic structure are nearly indistinguishable (Fig. S6). Therefore, a 5% uniaxial compressive strain along the *c*-axis was selected for calculations.

**Defect Formation Energy**

The formation energy of a defect X in charge state q was calculated using the following equation:

$E^{f}[X^{q}]=E_{\text{tot}}[X^{q}]-E_{\text{tot}}[\text{bulk}]-\sum_{i} n_{i}\mu_{i}+q(E_{F}+E_{VBM})+E_{\text{corr}}$

Here, $E_{\text{tot}}[X^{q}]$ is the total energy of the supercell containing defect X in charge state q, $E_{\text{tot}}[\text{bulk}]$ is the total energy of the perfect supercell, $n_{i}$ denotes the number of atoms of species i added ($n_{i}>0$) or removed ($n_{i}<0$) to form the defect, and $\mu_{i}$ is the corresponding chemical potential. $E_{F}$ represents the Fermi level relative to the valence band maximum (VBM) $E_{VBM}$, and $E_{\text{corr}}$ is a correction term accounting for finite k-point sampling effects and elastic/electrostatic interactions between periodic images for shallow impurities.

**Thermodynamic Transition Levels**

To rigorously characterize defect behavior as shallow or deep, the thermodynamic charge transition level $\epsilon(q_{1}/q_{2})$ was computed. This level corresponds to the Fermi energy at which the formation energies of defect X in charge states $q_{1}$ and $q_{2}$ are equal:

$$\epsilon(q_{1}/q_{2})=\frac{E^{f}[X^{q_{1}};E_{F}=0]-E^{f}[X^{q_{2}};E_{F}=0]}{q_{2}-q_{1}}$$

**Optical Transitions**

The normalized luminescence line shape is given, to leading order, by $G(\hbar\omega)=C\omega^{3}A(\hbar\omega)$, where $G(\hbar\omega)$ is the normalized spectral function and

$$A(\hbar\omega)=\sum_{m,n} w_{m}(T){\mid\langle\chi_{em}\mid\chi_{gn}\rangle\mid}^{2}\delta(E_{\text{ZPL}}+\hbar\omega_{em}-\hbar\omega_{gn}-\hbar\omega).$$

The constant $C$ normalizes the spectrum such that $C^{-1}=\int A(\hbar\omega)\omega^{3}d(\hbar\omega)$. The sum runs over all vibrational levels with energies $\hbar\omega_{em}$ and $\hbar\omega_{gn}$ of the excited and ground states, respectively. Here, $w_{m}(T)$ is the thermal occupation factor of the m-th vibrational level in the excited state, $\chi_{em}$ and $\chi_{gn}$ represent the ionic wavefunctions, and $E_{\text{ZPL}}$ is the zero-phonon line energy. This formulation adopts the Franck–Condon approximation, which assumes the electronic transition dipole moment is independent of ionic coordinates. We further assume that optical transitions originate from delocalized charge carriers and that excitonic effects are negligible**.**

**Defect and Charge Carrier Concentrations**

The self-consistent Fermi level was determined iteratively by imposing charge neutrality:

$$\sum_{D,q} qC_{D,q}-n_{0}+p_{0}=0,$$

where $C_{D,q}$ is the concentration of defect D in charge state q, and $n_{0}$ and $p_{0}$ are the free electron and hole concentrations, respectively.

The defect concentration is given by

$$C_{D,q}=gN_{D}\exp(-\frac{E_{D,q}}{k_{B}T_{\text{anneal}}}),$$

Where g is a degeneracy factor accounting for spin and configurational degrees of freedom, $N_{D}$ is the number of available defect sites per unit volume, $k_{B}$ is the Boltzmann constant, and $T_{\text{anneal}}$ is the annealing or synthesis temperature of the host material.

The free electron and hole concentrations are computed as:

$$n_{0}=\int_{E_{\text{CBM}}}^{\infty} \rho(E)f(E)dE,p_{0}=\int_{-\infty}^{E_{\text{VBM}}} \rho(E)[1-f(E)]dE,$$

where $\rho(E)$ is the density of states per unit volume and $f(E)$ is the Fermi–Dirac distribution:

$$f(E)=\frac{1}{\exp(\frac{E-E_{F}}{k_{B}T})+1}.$$

Here, $E_{F}$ is the Fermi level and *T* is the temperature.

**Molecular Dynamics Simulations**

For non-adiabatic molecular dynamics (NAMD) simulations, we first generated an adiabatic molecular dynamics (MD) trajectory. The system was equilibrated at 300 K for 6 ps in the canonical (NVT) ensemble. Subsequently, a 6 ps *ab initio* MD (AIMD) simulation was performed in the microcanonical (NVE) ensemble using a 1 fs time step for nuclear motion. Non-adiabatic coupling elements were extracted from the AIMD trajectories. To investigate the electron–phonon coupling between the luminescence center and the conduction band minimum (CBM), we analyzed the Fourier transform spectra of the energy shifts in the initial and final electronic states.

The parabolic band dispersion near the conduction band minimum (CBM) implies light effective mass and high electron mobility, whereas the flat bands at the valence band maximum (VBM) lead to heavy holes and low hole mobility. The VBM and CBM of Al_2_O_3_ are predominantly contributed by the O-3p and Al-4s orbitals, respectively.

**Chemical potentials.** In order to determine the stable region of the elemental chemical potentials (Δμ_Al_, Δμ_O,_ Δμ_Cr_), where each Δμ_i_ represents the chemical potential relative to the standard chemical potential (i.e., μ_Al_, μ_O_, and μ_Cr,_ respectively). The sum of μ_Al_, μ_O_, and μ_Cr_ is given by the formation energy of Al_2_O_3_ and Al_2_O_3_:Cr^3+^, ΔE_f_ (Al_2_O_3_) and ΔE_f_ (Al_31_O_48_Cr_1_), namely,

2Δμ_Al_ + 3Δμ_O_ = ΔE_f_ (Al_2_O_3_)

31Δμ_Al_ + 48Δμ_O_ + Δμ_Cr_ = ΔE_f_ (Al_31_O_48_Cr_1_)

In addition, Δμ_Zn_ and Δμ_S_ should not stabilize other competing phases such as Al, O_2_ and Cr_2_O_3_, namely,

Δμ_Al_ ≤ 0

Δμ_O_ ≤ 0

2Δμ_Cr_ + 3Δμ_O_ ≤ ΔE_f_ (Cr_2_O_3_)

Such constraints determine the chemical potential ranges for Δμ_Al_, Δμ_O,_ Δμ_Cr_ that stabilize Al_2_O_3_ and Al_2_O_3_:Cr^3+^. Here, we get that when O-rich (Δμ_O_ = 0) with Δμ_Al_ = -3.94 eV and Δμ_Cr_ = -11.85 eV, while when Al-rich (Δμ_Al_ = 0) with Δμ_O_ = -2.62 eV and Δμ_Cr_ = -12.94 eV within the stable region of Al_2_O_3_:Cr^3+^.

**Defect Formation Energy and E_F_.** First, all intrinsic point defects in Al_2_O_3_ were considered, including two vacancies (V_Al_, V_O_), two interstitials (Al_i_, O_i_), two antisites (Al_O_, O_Al_) and two Frenkel defects (V_Al_+Al_i_, V_O_+O_i_), represented by Frenkel_Al and Frenkel_O, respectively.

**Formation of a Defect Complex.** All potential intrinsic point defects close to the Cr molecule were considered, including two vacancies (Cr_Al_+V_Al_, Cr_Al_+V_O_), two interstitials (Cr_Al_+Al_i_, Cr_Al_+O_i_), two antisites (Cr_Al_+Al_O_, Cr_Al_+O_Al_) and two Frenkel defects (Cr_Al_+V_Al_+Al_i_, Cr_Al_+V_O_+O_i_).

**Cr_Al_^0^/ Cr_Al_^1+^ charge transition mechanism dominate the self-recoverable ML.**

The effective concentration of Cr_Al_ centers reaches the order of ${10}^{26}\text{ }cm^{-3}$, whereas other defect species remain at approximately ${10}^{15}\text{ }cm^{-3}$, indicating a difference of more than ten orders of magnitude (Fig. 4a). Within a kinetic framework, the charge transition rate $R$can be expressed as

$$R=CN_{D}p,$$

where $N_{D}$is the defect concentration, *C* is the capture coefficient, and *p* represents the carrier density^5^. Since the luminescence intensity is closely related to the charge transition rate^6,7^, and assuming comparable capture cross sections for defect-assisted charge transfer, the transition rate is therefore dominated by the defect concentration. Consequently, the reversible Cr_Al_^0^/Cr_Al_^1+^ charge transition overwhelmingly governs the defect-mediated ML process as the dominant luminescent center, with the emitted light originating from the d-d electronic transitions of Cr dopants^8,9^, while contributions from other defect-related channels are statistically negligible.

**Reference.**

1. Eng, P. J. *et al.* Structure of the Hydrated α-Al_2_O_3_ (0001) Surface. *Science* **288**, 1029–1033 (2000).

2. Peng, Z., Chen, X., Fan, Y., Srolovitz, D. J. & Lei, D. Strain engineering of 2D semiconductors and graphene: from strain fields to band-structure tuning and photonic applications. *Light Sci Appl* **9**, 190 (2020).

3. Dymkowski, K. & Ederer, C. Strain-induced insulator-to-metal transition in LaTiO_3_ within DFT + DMFT. *Phys. Rev. B* **89**, 161109 (2014).

4. Park, H., Millis, A. J. & Marianetti, C. A. Influence of quantum confinement and strain on orbital polarization of four-layer LaNiO_3_ superlattices: A DFT+DMFT study. *Phys. Rev. B* **93**, 235109 (2016).

5. Alkauskas, A., Yan, Q. & Van De Walle, C. G. First-principles theory of nonradiative carrier capture via multiphonon emission. *Phys. Rev. B* **90**, 075202 (2014).

6. Alkauskas, A., Lyons, J. L., Steiauf, D. & Van De Walle, C. G. First-Principles Calculations of Luminescence Spectrum Line Shapes for Defects in Semiconductors: The Example of GaN and ZnO. *Phys. Rev. Lett.* **109**, 267401 (2012).

7. Lyons, J. L., Janotti, A. & Van De Walle, C. G. Shallow versus Deep Nature of Mg Acceptors in Nitride Semiconductors. *Phys. Rev. Lett.* **108**, 156403 (2012).

8. Liu, S. *et al.* Bright Chromium‐Sensitized Lanthanide NIR‐II Mechanoluminescence in a Piezoelectric Oxide. *Adv. Mater.* e06957 (2025).

9. Suo, H. *et al.* A broadband near-infrared nanoemitter powered by mechanical action. *Matter* **6**, 2935–2949 (2023).


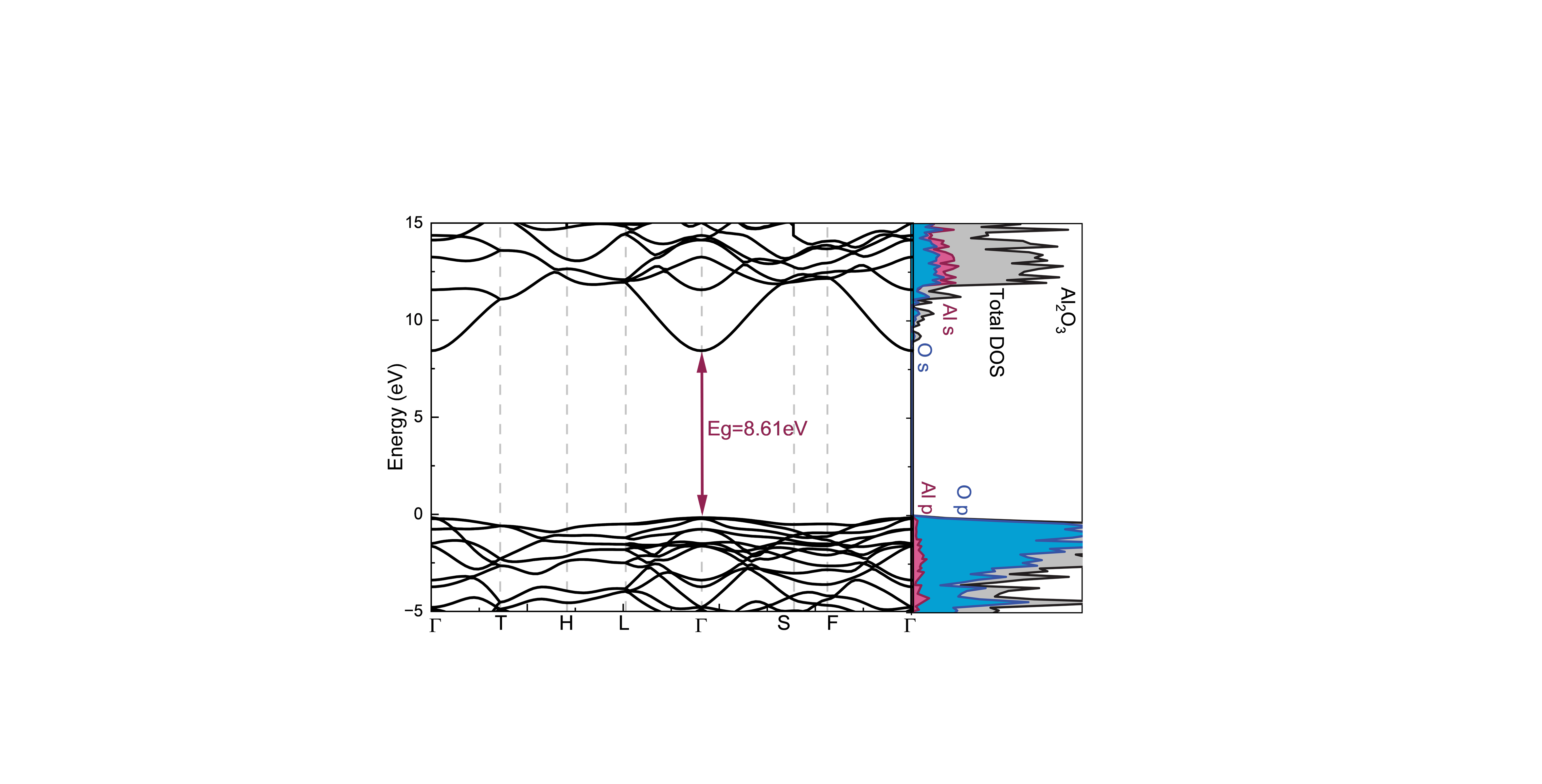


**Fig. S1 | Electronic state structure** **of Al_2_O_3_.** Electronic band structure and electronic density of states of Al_2_O_3_.


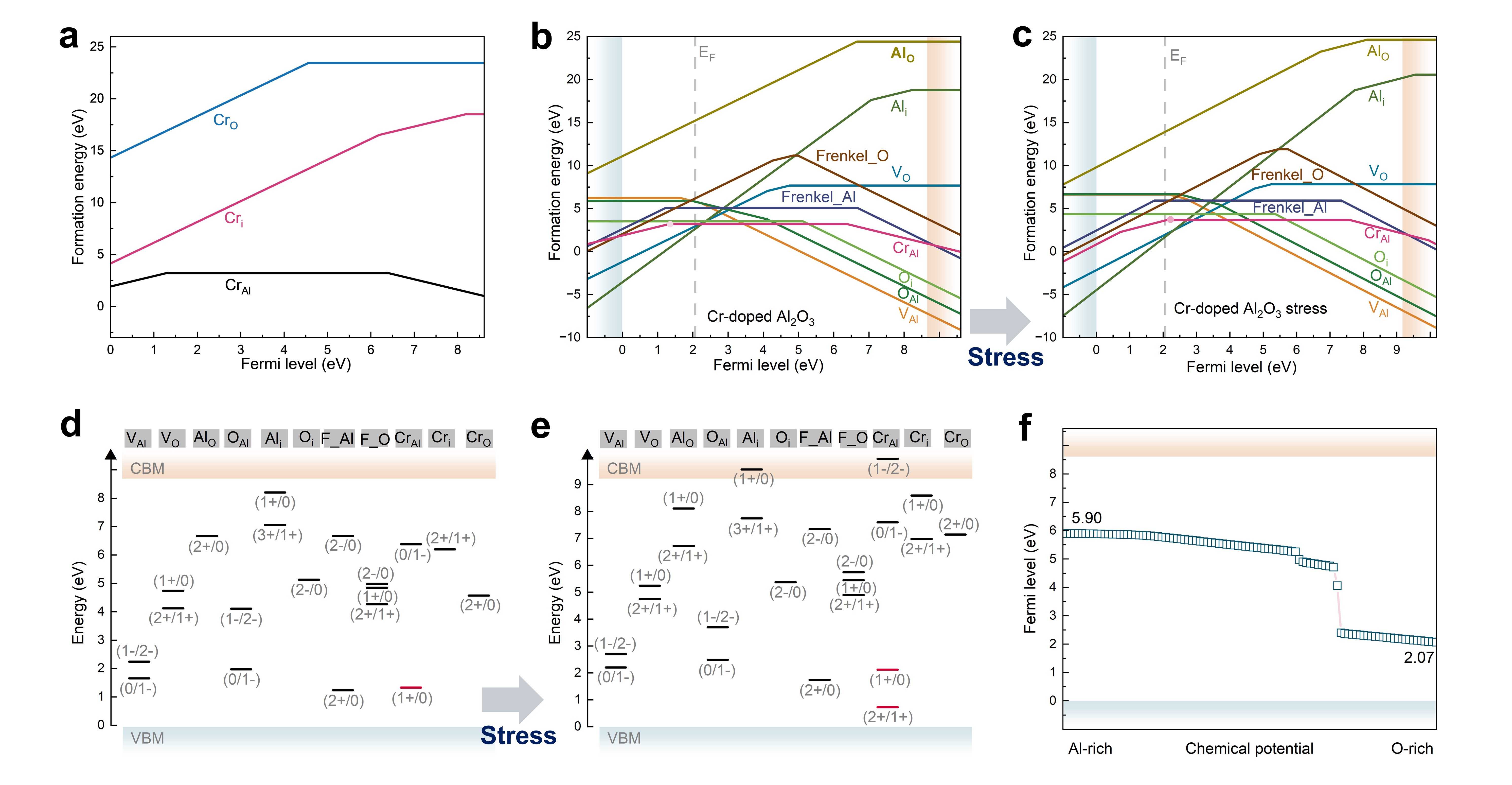


**Fig. S2 | Electronic state structure and thermodynamic properties involved in ML. a** Calculated formation energies of Al_2_O_3_:Cr^3+^. **b** Calculated formation energies of Al_2_O_3_:Cr^3+^ without stress. **c** Calculated formation energies of Al_2_O_3_:Cr^3+^ with stress. **d** Charge state transition levels of point defects in Al_2_O_3_:Cr^3+^ without stress. **e** Charge state transition levels of point defects in Al_2_O_3_:Cr^3+^ with stress. **f** Self-consistent Fermi level at 300 K in Al_2_O_3_:Cr^3+^ crystals grown at 1 950 K as a function of the growth condition.


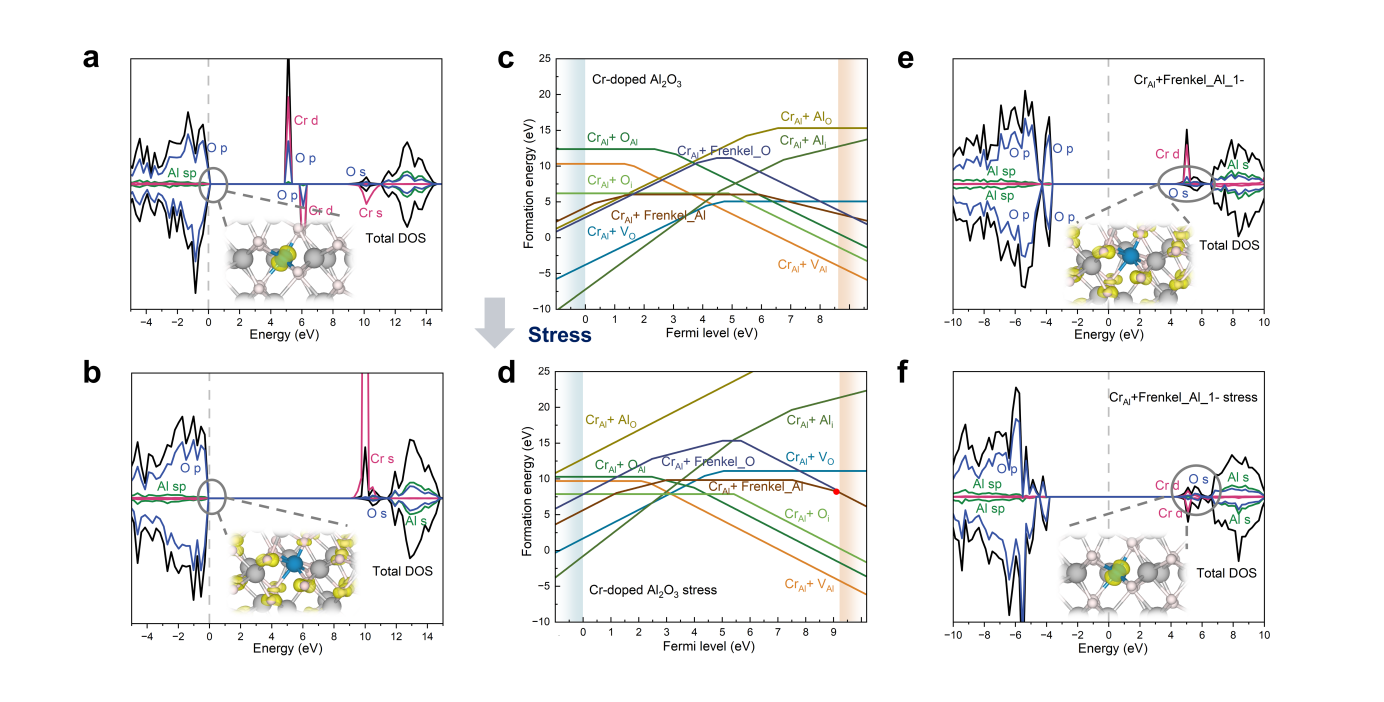


**Fig. S3 | The origin of carriers in radiative recombination. a-b** Electronic DOS of Cr_Al_^2+^ and the highest occupied state partial charge density that represent **(a)** without stress and **(b)** with stress. **c-d** Calculated formation energies of intrinsic and extrinsic point defects considered simultaneously formed the complexes in Al_2_O_3_:Cr^3+^ under S-rich that represent **(c)** without stress and **(d)** with stress. **e-f** Electronic DOS of (Cr_Al_+Frenkel_Al) and the lowest unoccupied state partial charge density that represent **(e)** without stress and **(f)** with stress.


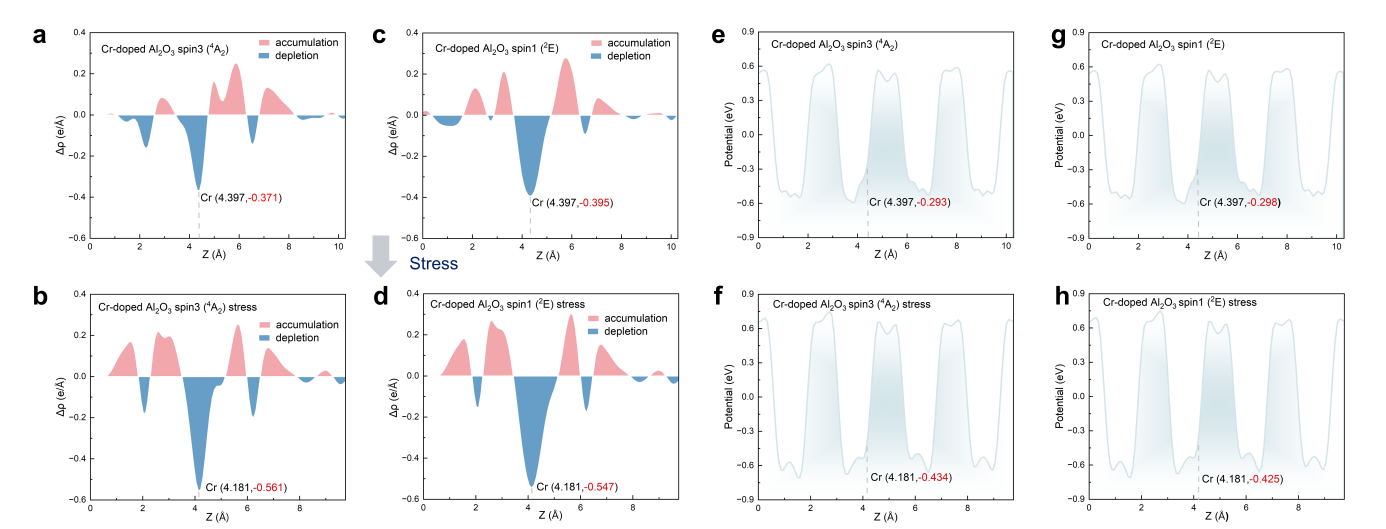


**Fig. S4 | Driving force for carrier migration. a-b** Plane-averaged charge difference along the vertical z-direction to the Al_2_O_3_ in ground state. Red (blue) region represents charge accumulation (depletion) regions. **(a)** without stress and **(b)** with stress. **c-d** Plane-averaged charge difference along the vertical z-direction to the Al_2_O_3_ in excited state. Red (blue) region represents charge accumulation (depletion) regions. **(c)** without stress and **(d)** with stress. **e-f** Effective potential profile of Al_2_O_3_ in ground state. **(e)** without stress and **(f)** with stress. **g-h** Effective potential profile of Al_2_O_3_ in excited state. **(g)** without stress and **(h)** with stress.


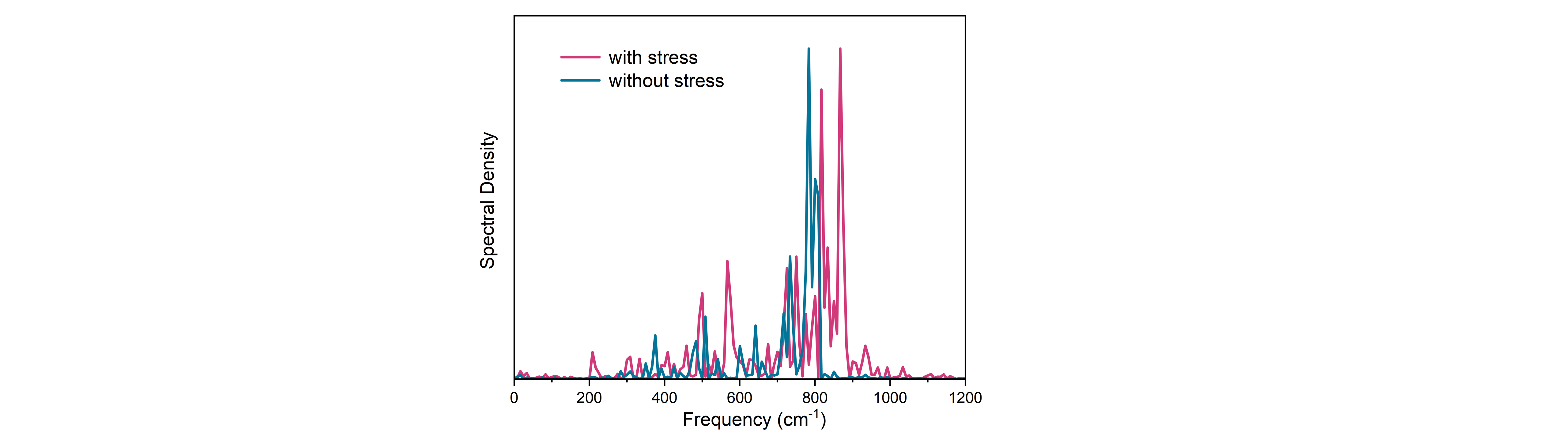


**Fig. S5 | Driving Forces for Charge Carriers.** Electron-phonon coupling strength in the Al_2_O_3_:Cr^3+^ system with and without stress.


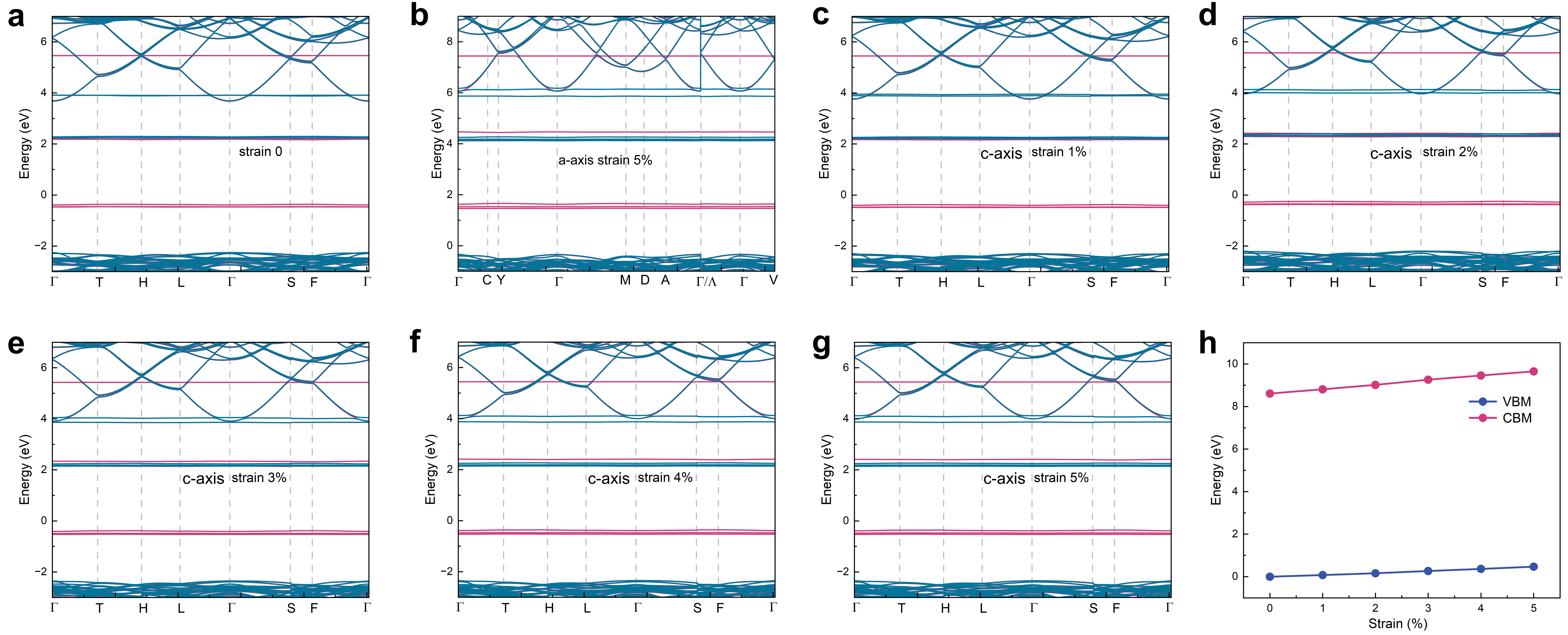


**Fig. S6 | Band bending under stress facilitates charge exchange between defects and band edges. a** Calculated band gap of Al_2_O_3_:Cr^3+^ without the external strain. **b** Calculated band gap of Al_2_O_3_:Cr^3+^ with 5% external strain along the a-axis. **c-g** Calculated band gap of Al_2_O_3_:Cr^3+^ with the external strain along the c-axis increasing from 1% to 5%. **h** Energy levels of VBM and CBM in Al_2_O_3_ under different strains along the c-axis.


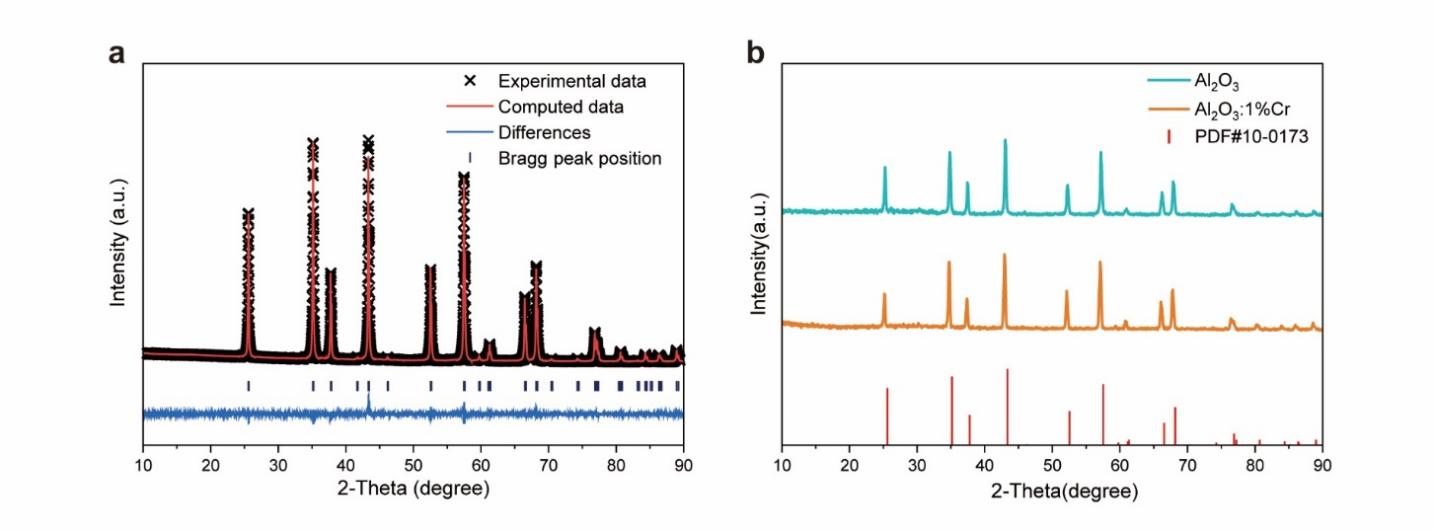


**Fig. S7 | a** Rietveld refinement of Al_2_O_3_. **b** XRD patterns of Al_2_O_3_:1%Cr^3+^ and undoped Al_2_O_3_.


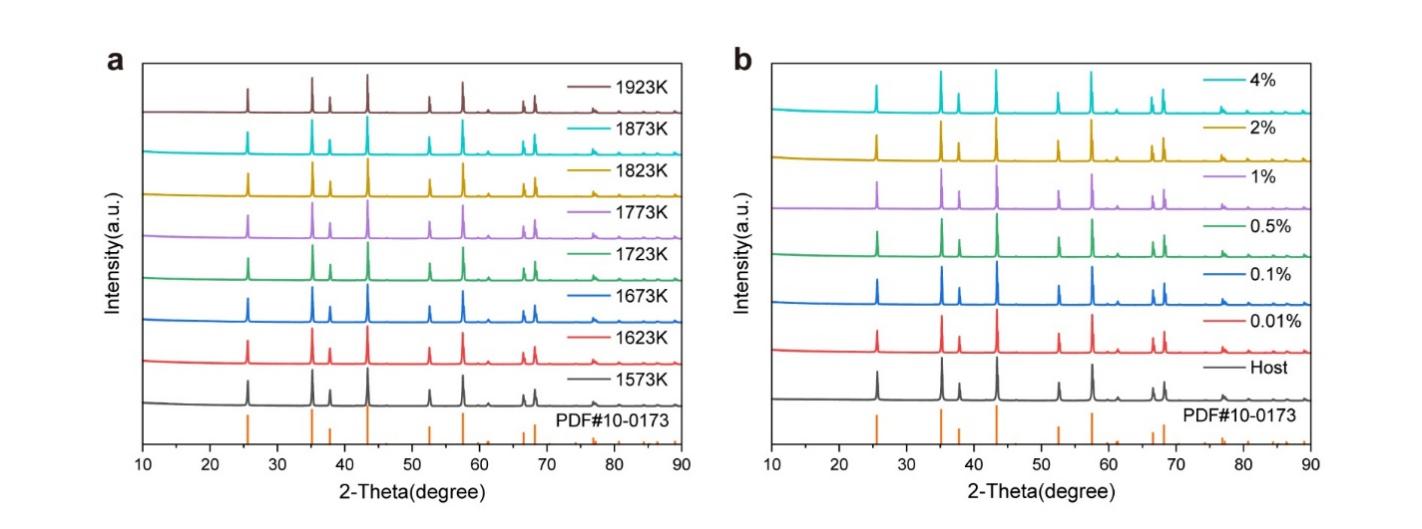


**Fig. S8** **| a** XRD patterns of Al_2_O_3_:1%Cr^3+^ samples synthesized at different annealing temperatures (1 573–1 923 K). **b** XRD patterns of Al_2_O_3_:xCr^3+^ samples with varying Cr^3+^ concentrations (x = 0.01–4 %) annealed at 1 923 K.


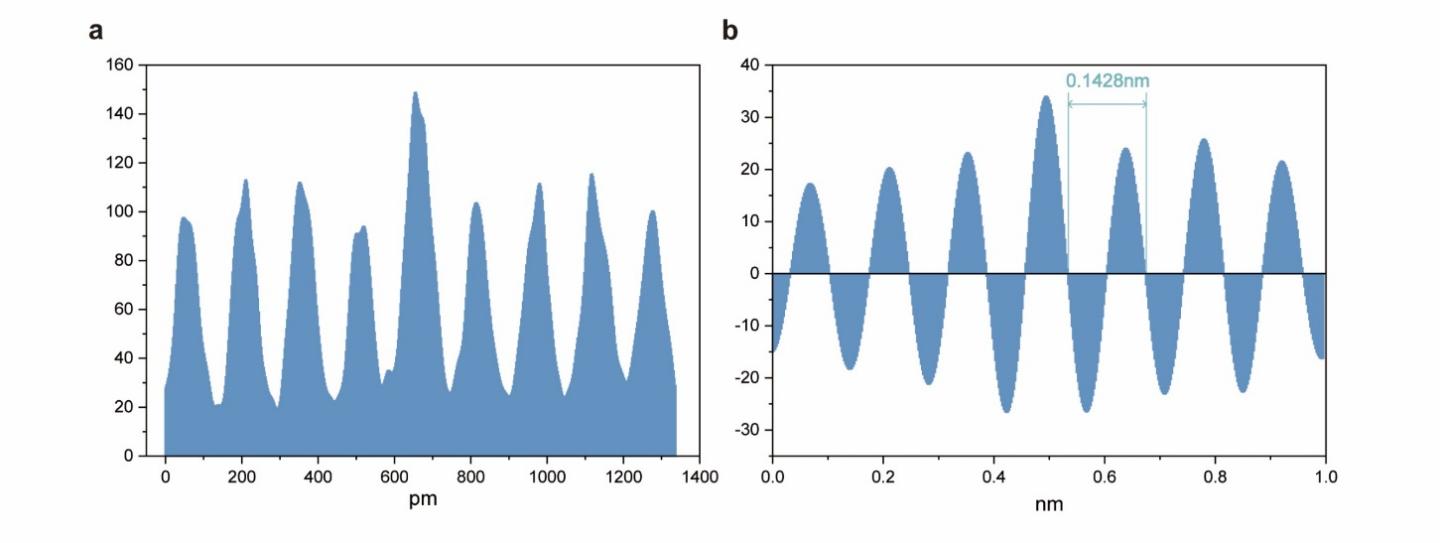


**Fig. S9 |** **a** Brightness-enhanced area scan of the light-blue region in Fig 2c, indicating the incorporation of Cr^3+^ ions due to their stronger electron scattering compared to Al. **b** Line profile taken along the light-blue line in Fig 2d, revealing an interplanar spacing of 0.1428 nm.


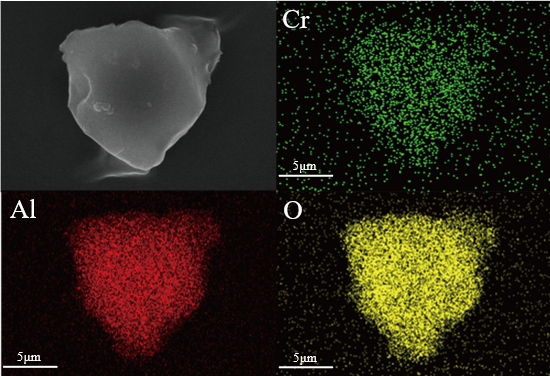


**Fig. S10 |** SEM and EDS images of Al_2_O_3_:4%Cr^3+^ sample.


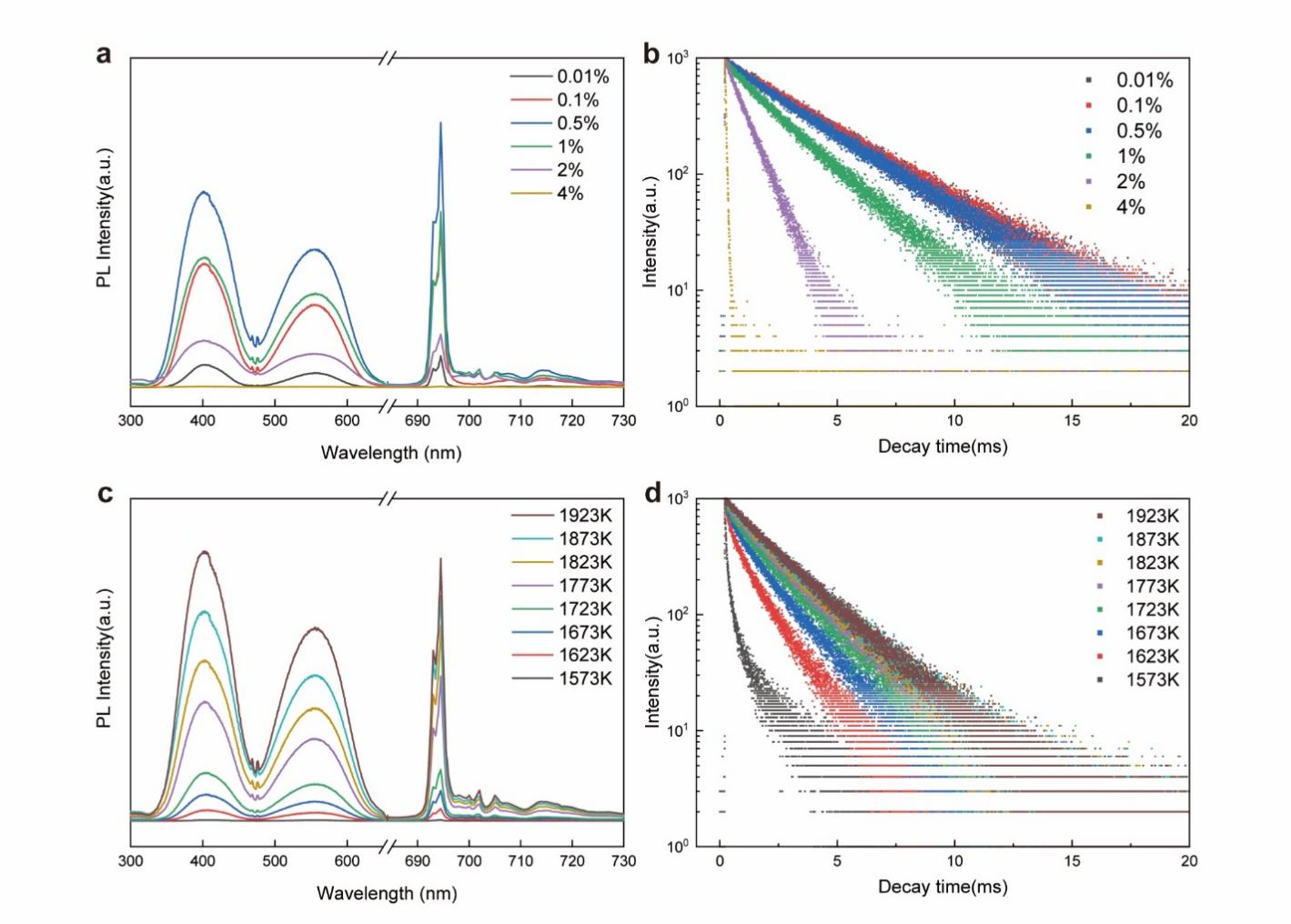


**Fig. S11 | a** PLE and PL spectra of Al_2_O_3_:xCr^3+^ samples with varying doping concentrations (x = 0.01–4 %). **b** Fluorescence lifetimes of Al_2_O_3_:xCr^3+^ samples with different doping concentrations (x = 0.01–4 %). **c** PLE and PL spectra of Al_2_O_3_:1%Cr^3+^ samples annealed at different temperatures (1 573–1 923 K). **d** Fluorescence lifetimes of Al_2_O_3_:1%Cr^3+^ samples annealed at various temperatures (1 573–1 923 K).


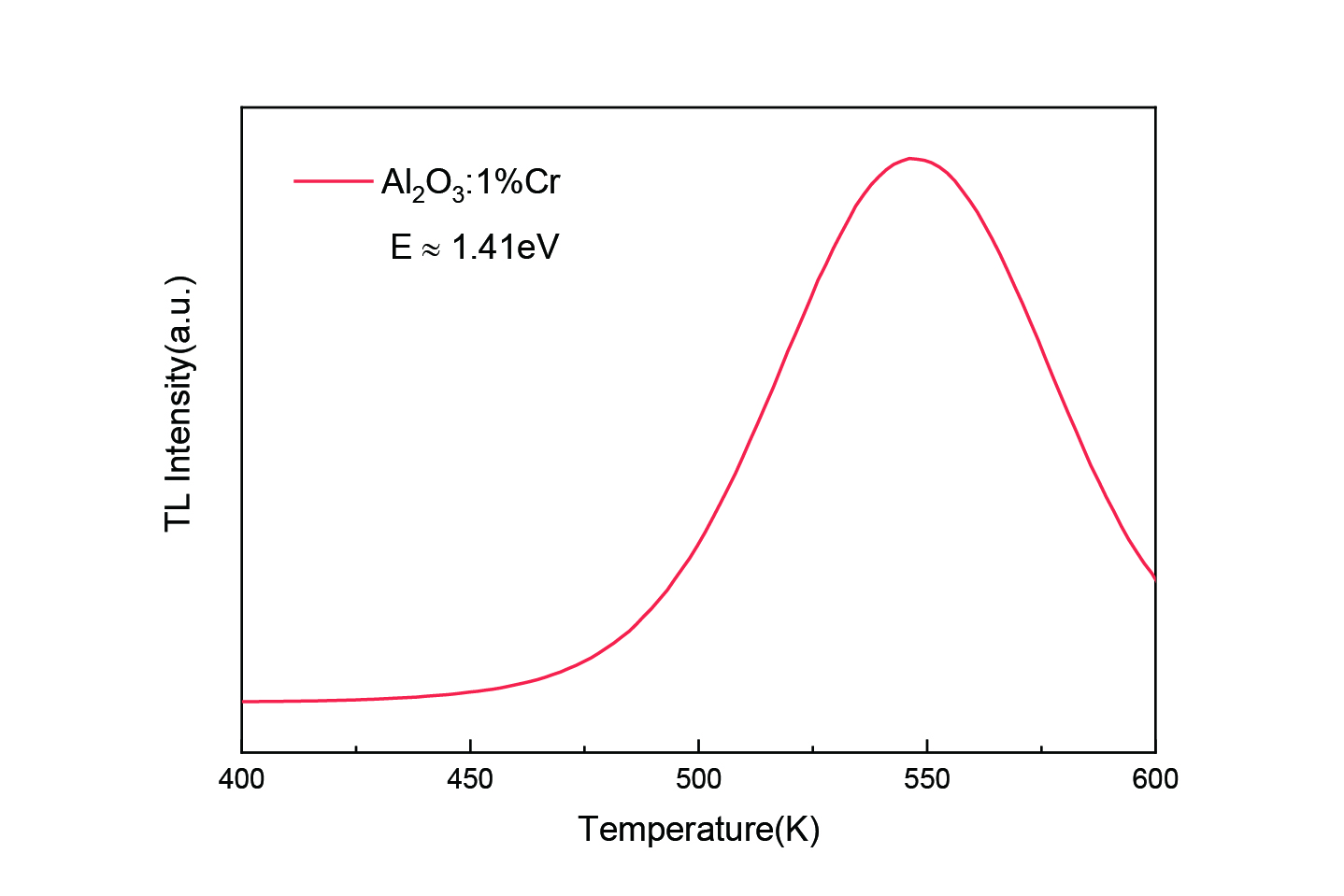


**Fig. S12 |** TL glow curve of the Al_2_O_3_:Cr^3+^ sample, showing a dominant emission peak centered at approximately 546 K.


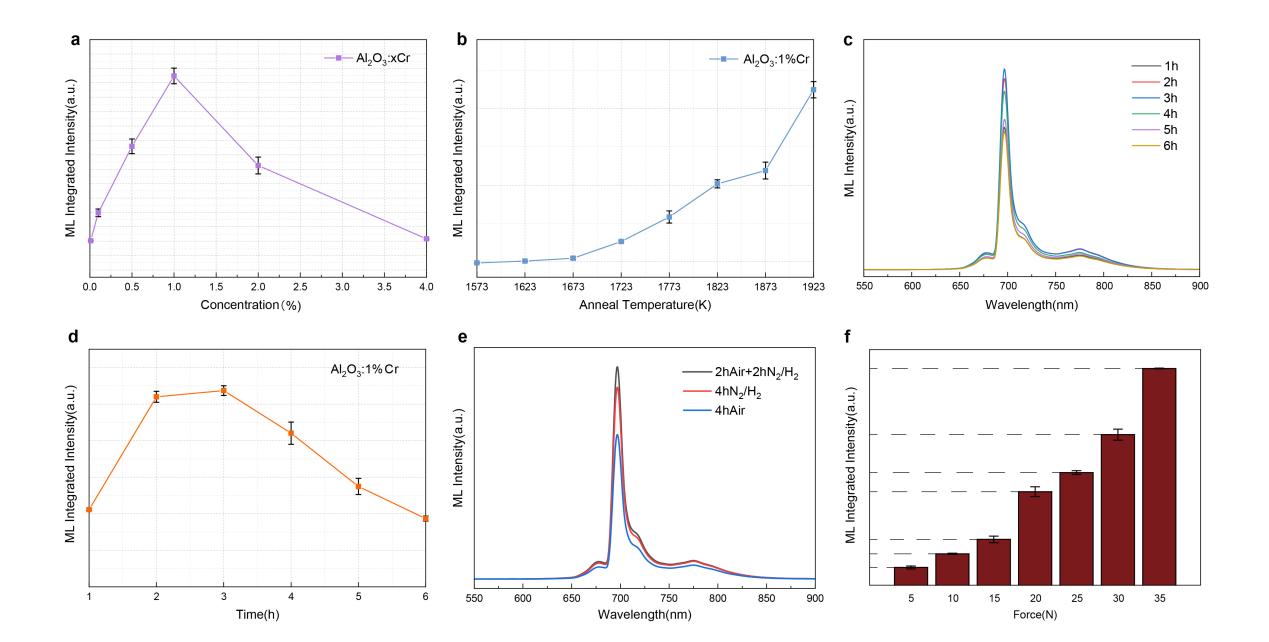


**Fig. S13|** **a** ML Integrated intensity of Al_2_O_3_:Cr^3+^ with varying dopant concentrations. **b** ML Integrated intensity of Al_2_O_3_:1%Cr^3+^ annealed at different temperatures. **c** ML spectra of Al_2_O_3_:1%Cr^3+^ samples annealed at different holding durations (1–6 h). **d** ML Integrated intensity of Al_2_O_3_:1%Cr^3+^ with varying holding durations. **e** ML spectra of Al_2_O_3_:1%Cr^3+^ after annealing under different atmospheres: 4 h in N_2_/N_2_, 2 h in air followed by 2 h in N_2_/N_2_, and 4 h in air. **f** ML integrated intensity of Al_2_O_3_:Cr^3+^ as a function of applied force, showing a near-linear relationship.


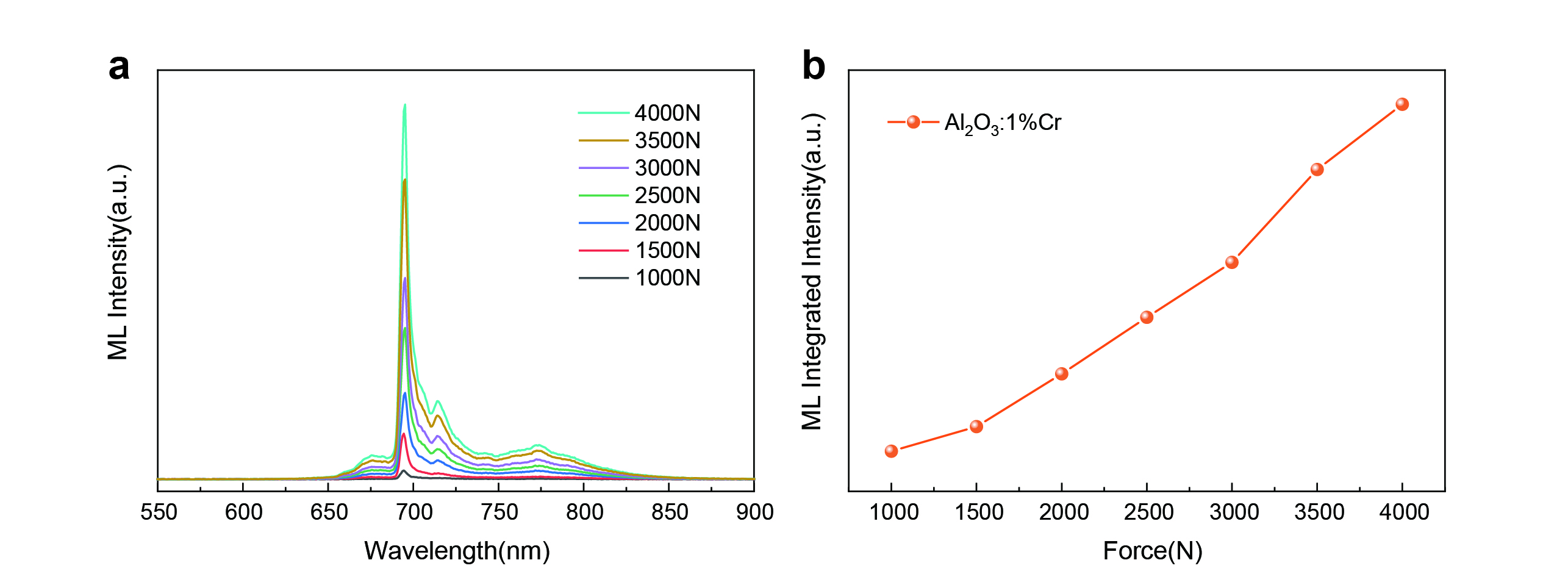


**Fig. S14 | a** Near-infrared ML spectra of Al_2_O_3_:Cr^3+^ under high applied loads from 1000 N to 4000 N in 500 N increments. **b** Integrated ML intensity as a function of applied load.


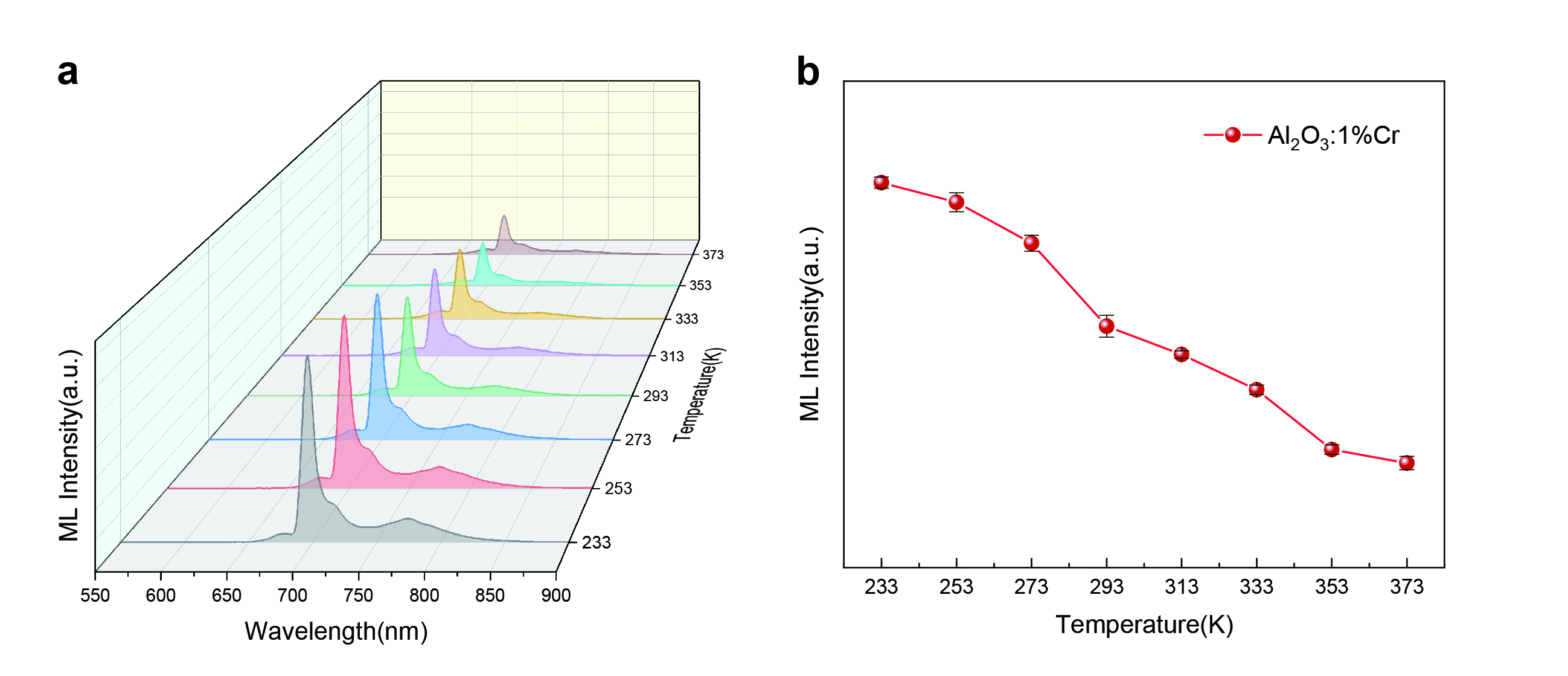


**Fig. S15 | a** Temperature-dependent ML spectra of Al_2_O_3_:Cr^3+^ measured from 233 to 373 K. **b** Corresponding evolution of the integrated ML intensity as a function of temperature.


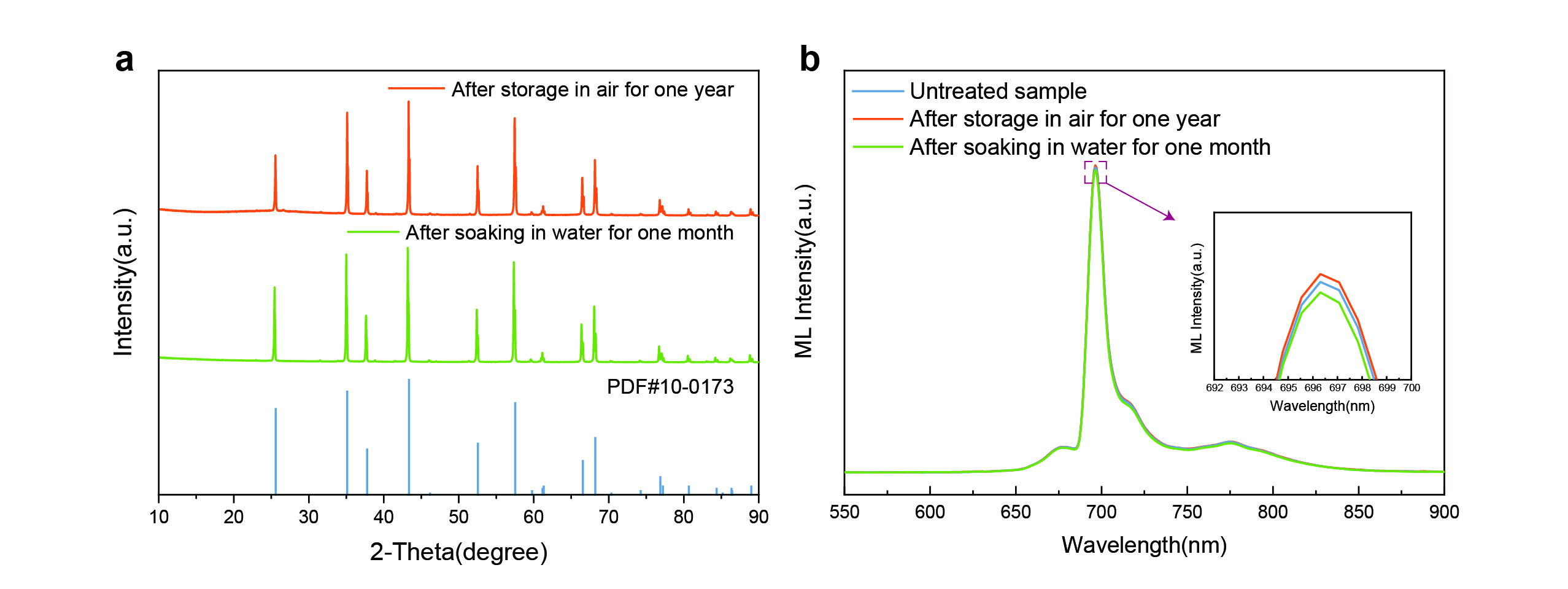


**Fig. S16 | Structural and ML stability of Al_2_O_3_:Cr^3+^ after long-term air storage and water immersion.** **a** XRD patterns. **b** ML intensity comparison.


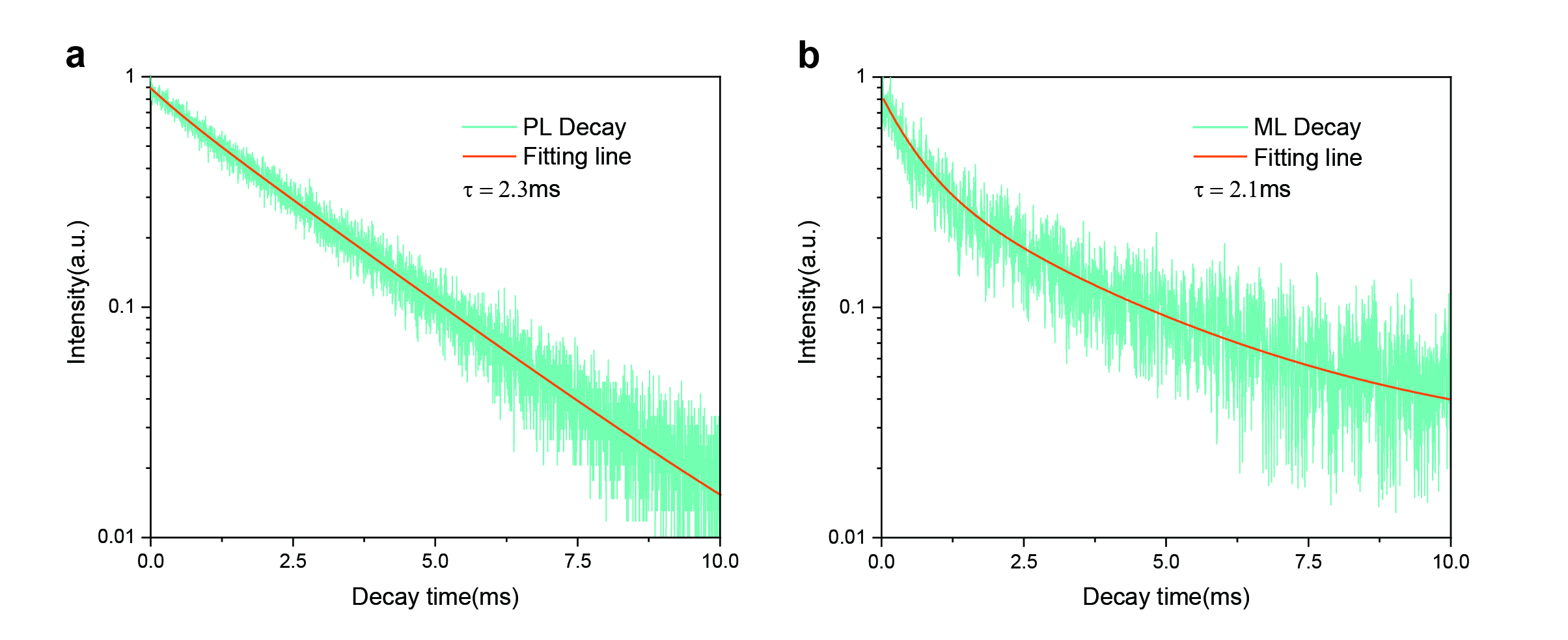


**Fig. S17 |** PL **(a)** and ML (**b)** decay curve of Al_2_O_3_:Cr^3+^.


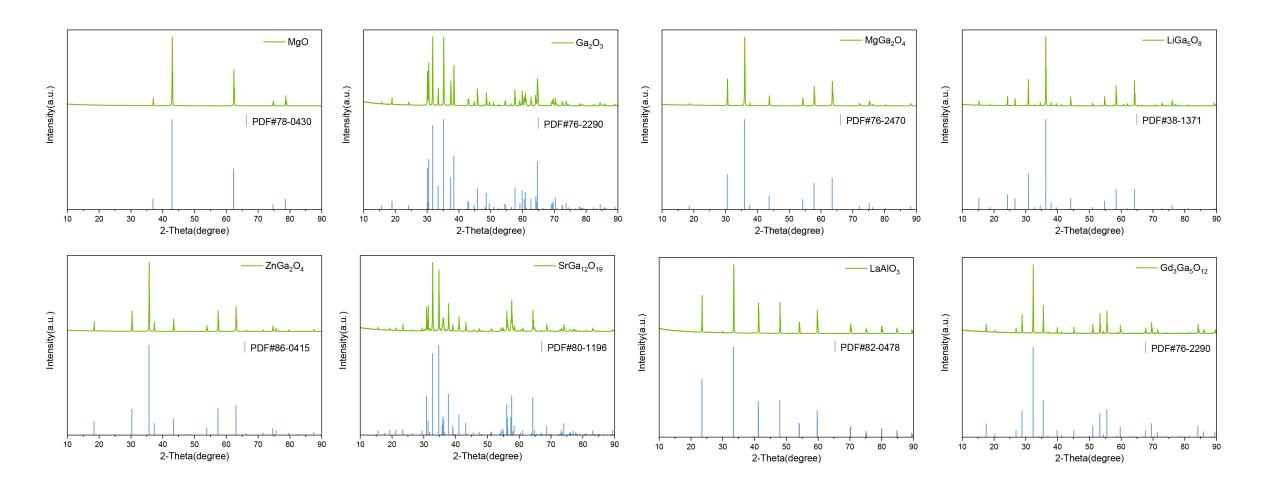


**Fig. S18 |** XRD patterns of previously reported Cr^3+^-doped material systems.


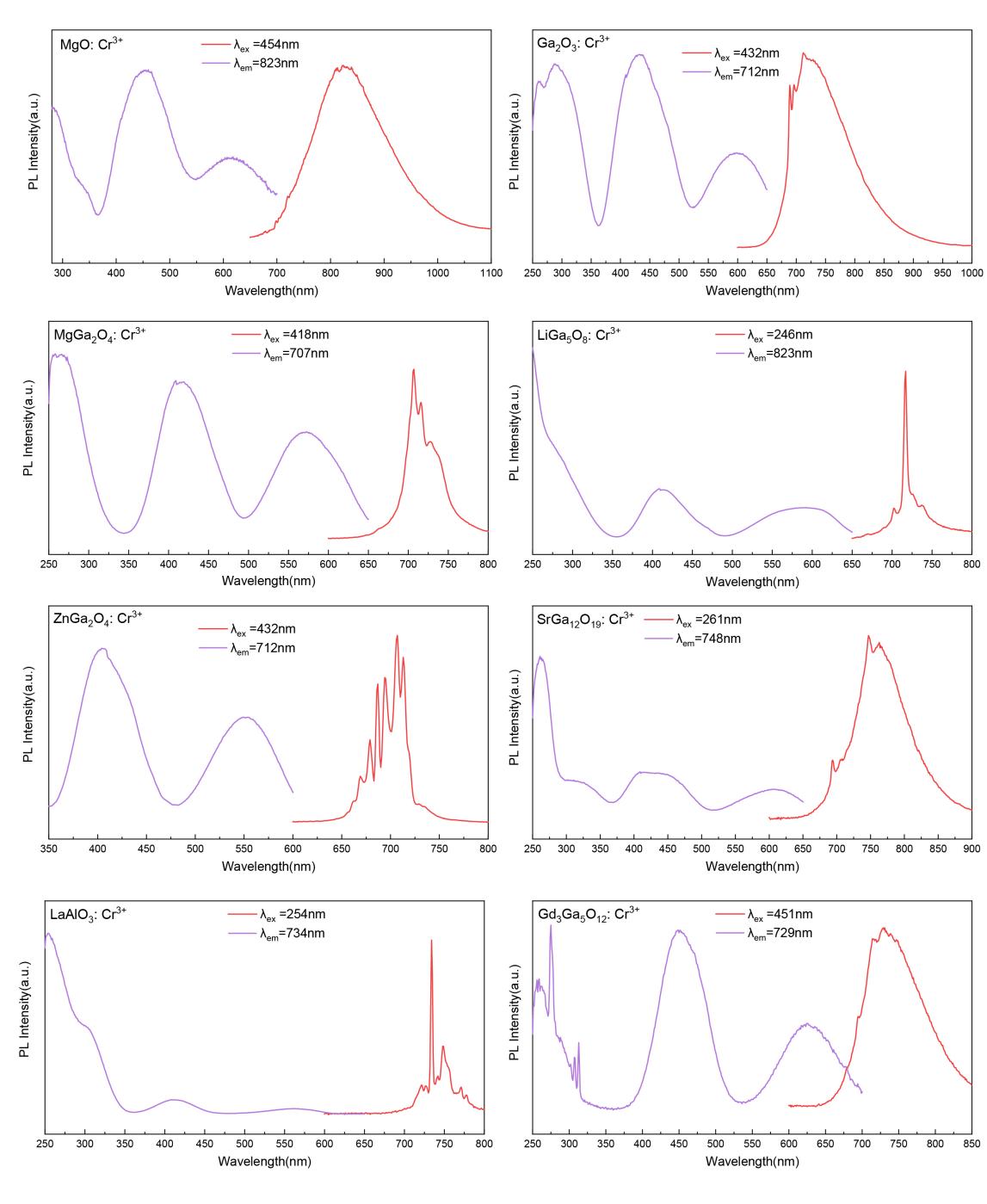


**Fig. S19 |** PL spectra of previously reported Cr^3+^-doped material systems.


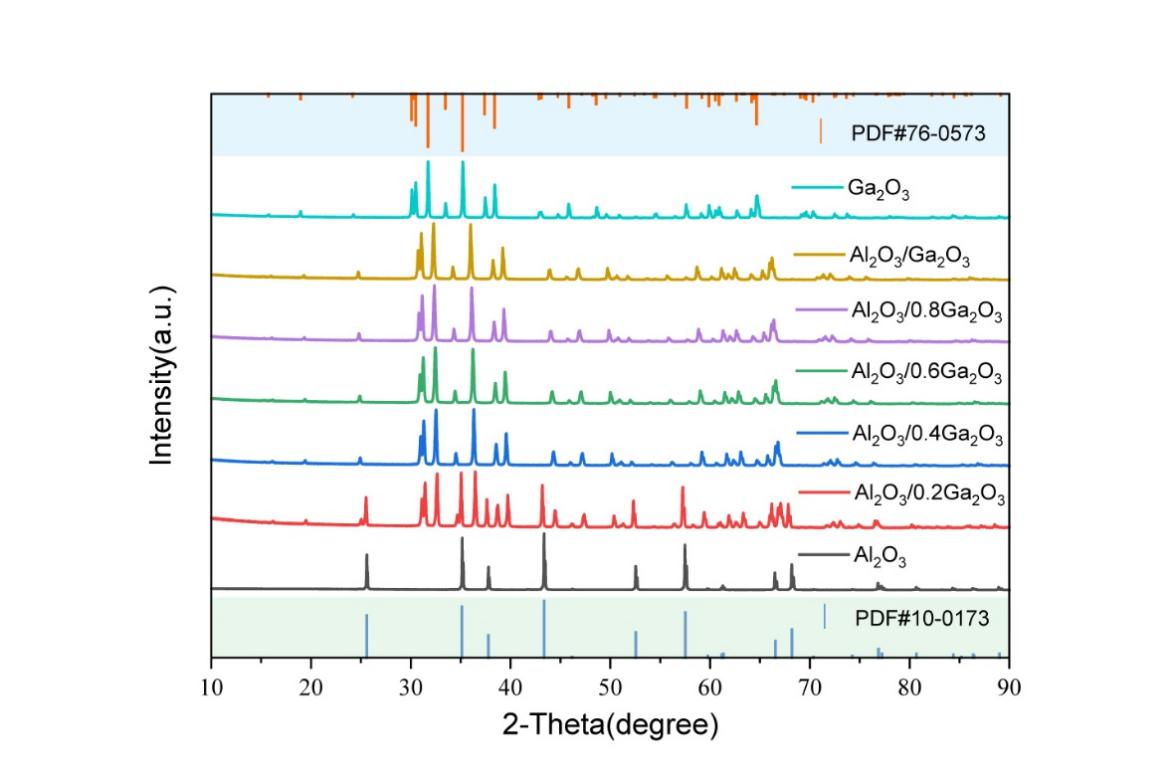


**Fig. S20 |** XRD patterns of Al_2_O_3_/Ga_2_O_3_:Cr^3+^ heterostructured materials with different composition ratios.

**Table S1 |** Excitation peaks, emission peaks, and FWHM of Al_2_O_3_/Ga_2_O_3_:Cr^3+^ heterojunction materials.

| **Al_2_O_3_/Ga_2_O_3_** | **Ex(nm)** | | **Em(nm)** | | | **FWHM** | |
| --- | --- | --- | --- | --- | --- | --- | --- |
|  | **Ⅰ** | **Ⅱ** | **Ⅰ** | **Ⅱ** | **Ⅲ** |  |  |
| 1:0 | 402 | 558 | - | 694 | - | 2 |  |
| 1:0.2 | 404 | 561 | 686 | 694 | 715 | 5 |  |
| 1:0.4 | 418 | 581 | 686 | 699 | 709 | 46 |  |
| 1:0.6 | 419 | 583 | 687 | 700 | 710 | 60 |  |
| 1:0.8 | 423 | 588 | 688 | 701 | 711 | 80 |  |
| 1:1 | 424 | 589 | 689 | 702 | 712 | 92 |  |
| 0:1 | 440 | 605 | 690 | 698 | 740 | 97 |  |

**Table S2 |** QY of Al_2_O_3_:Cr^3+^ and Al_2_O_3_/Ga_2_O_3_:Cr^3+^ heterojunction materials

| **Host** | **Concentration** | **Temperature** | **QY** |
| --- | --- | --- | --- |
| Al_2_O_3_ | 0.5% | 1650℃ | 94% |
| Al_2_O_3_ | 1% | 1650℃ | 92.6% |
| Al_2_O_3_ | 2% | 1650℃ | 70.8% |
| Al_2_O_3_ | 4% | 1650℃ | 9% |
| Al_2_O_3_ | 1% | 1550℃ | 84% |
| Al_2_O_3_ | 1% | 1450℃ | 54.2% |
| Al_2_O_3_ | 1% | 1350℃ | 23% |
| Al_2_O_3_/0.2Ga_2_O_3_ | 1% | 1650℃ | 89.8% |
| Al_2_O_3_/0.4Ga_2_O_3_ | 1% | 1650℃ | 85.8% |
| Al_2_O_3_/0.6Ga_2_O_3_ | 1% | 1650℃ | 84.4% |
| Al_2_O_3_/0.8Ga_2_O_3_ | 1% | 1650℃ | 82.6% |
| Ga_2_O_3_ | 1% | 1650℃ | 80.2% |


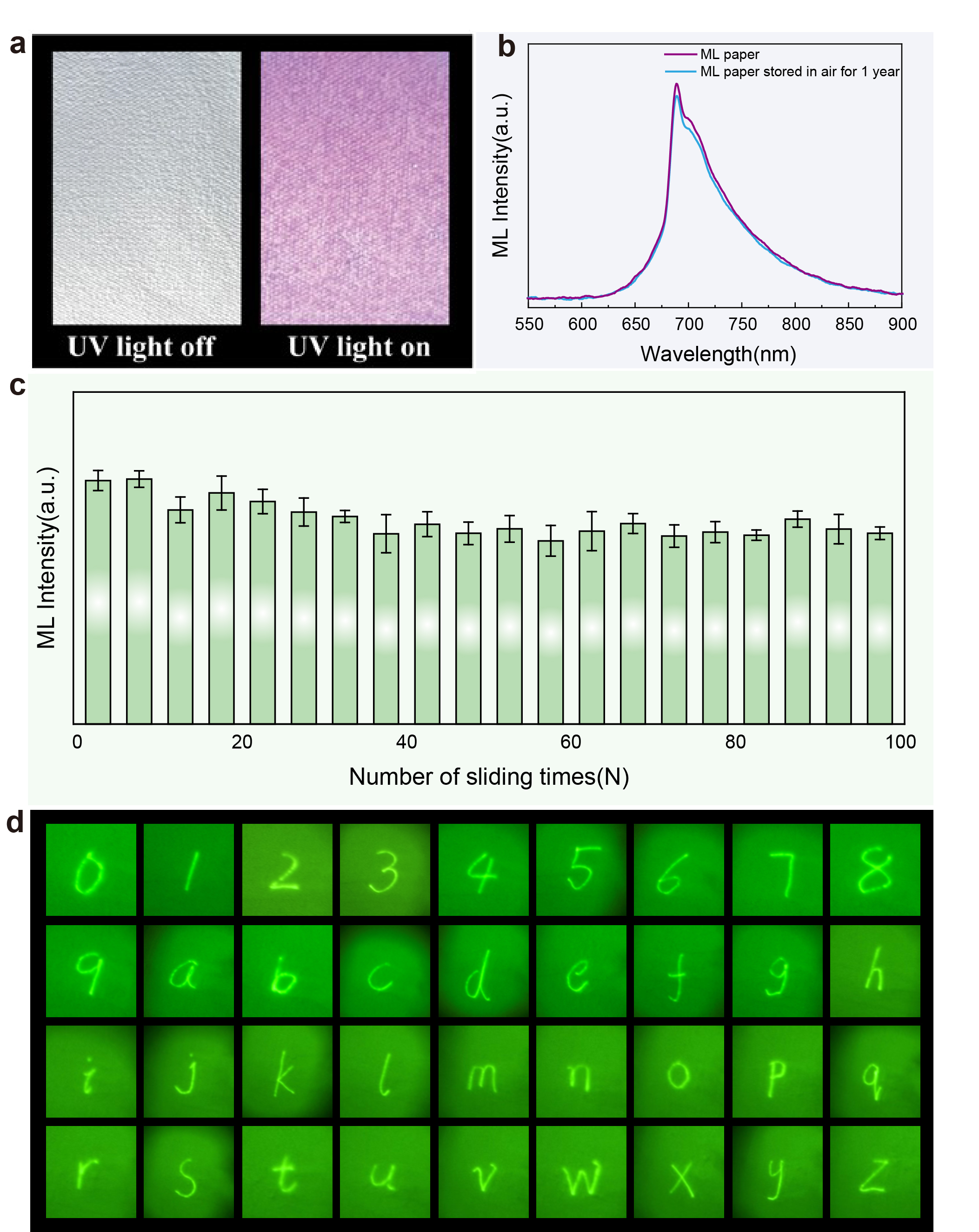


**Fig. S21 | a** Photographs of the ML paper under 365 nm UV light in the on and off states. **b** ML emission of the ML paper after 1 year natural storage under ambient conditions. **c** ML intensity of the ML paper during repeated mechanical loading–unloading cycles. **d** Handwritten luminescent trails on ML paper forming digits (1–9) and letters (a–z).


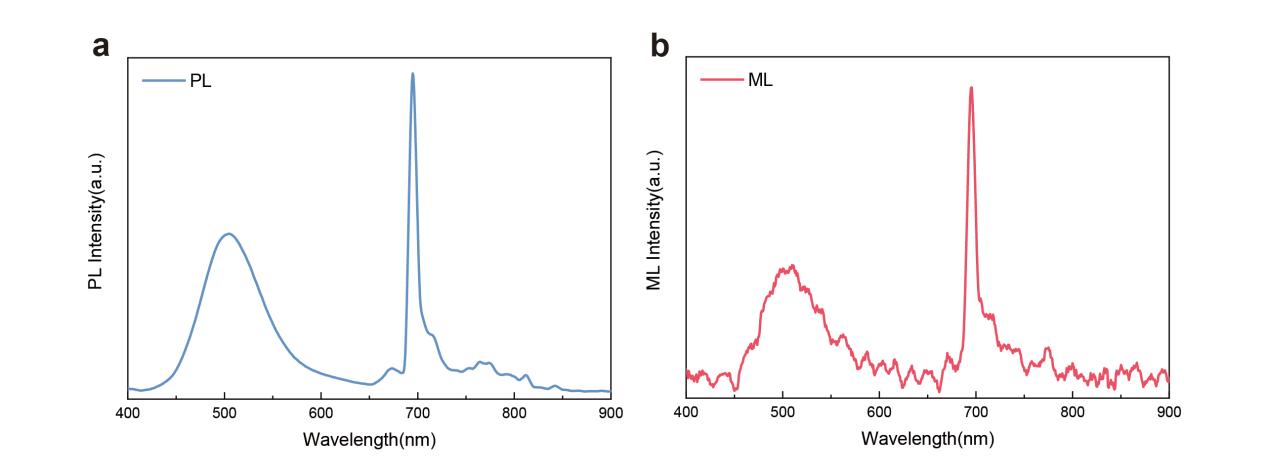


**Fig. S22 |** PL spectra **(a)** and ML spectra **(b)** of Al_2_O_3_:Cr^3+^/ZnS:Cu^+^-based PDMS.


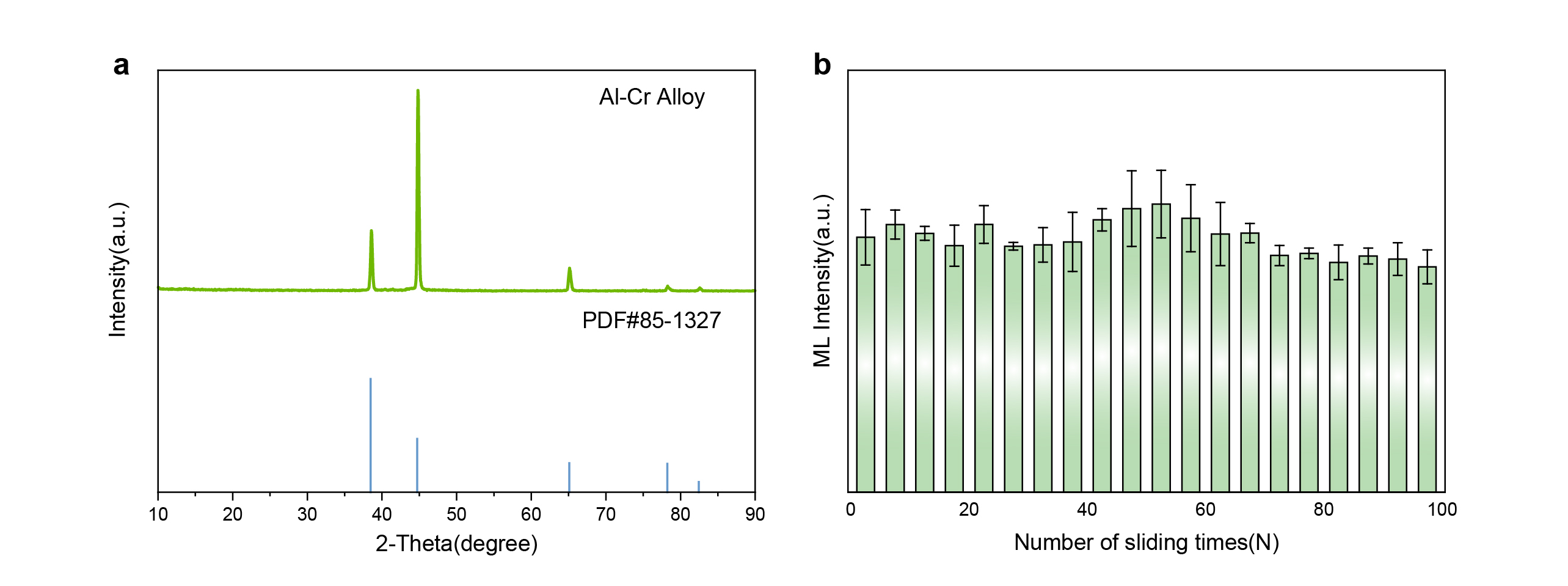


**Fig. S23 |** **a** XRD pattern of the Cr–Al alloy. **b** ML intensity of the Cr–Al alloy during repeated mechanical loading–unloading cycles.


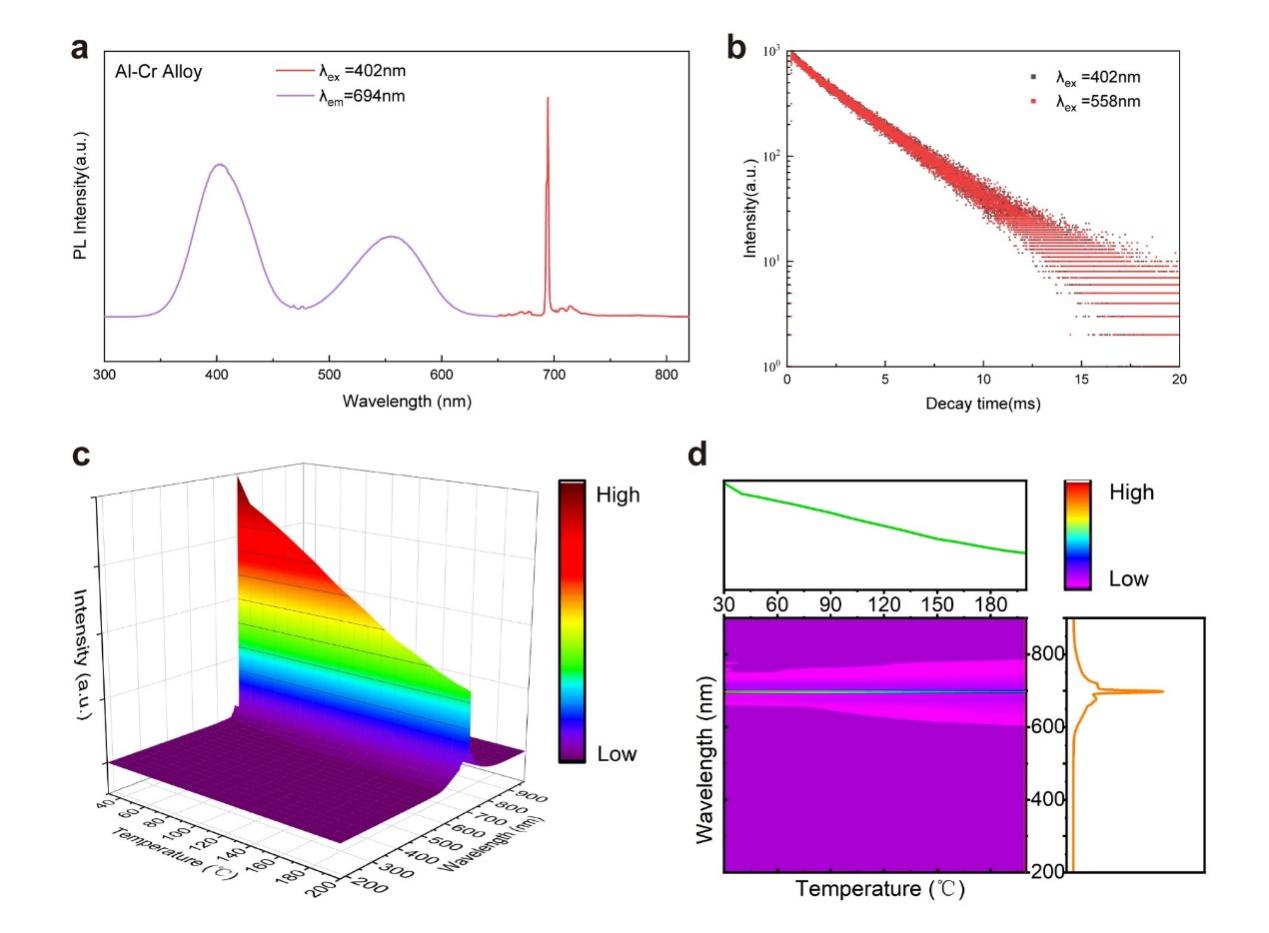


**Fig. S24 | a** PL spectrum of the Al_2_O_3_:Cr^3+^ thin layer formed on the Cr–Al alloy surface. **b** lifetime monitored at 402 nm and 558 nm excitation at 694 nm. 3D plots **(c)** and 2D plots **(d)** of the PL at different temperatures.
